# Oral health‐related quality of life of patients rehabilitated with fixed and removable implant‐supported dental prostheses

**DOI:** 10.1111/prd.12419

**Published:** 2022-02-01

**Authors:** Ho‐Yan Duong, Andrea Roccuzzo, Alexandra Stähli, Giovanni E. Salvi, Niklaus P. Lang, Anton Sculean

**Affiliations:** ^1^ Department of Periodontology School of Dental Medicine University of Bern Bern Switzerland

**Keywords:** dental implants, esthetics, patient‐reported outcome measures, patient satisfaction, quality of life

## Abstract

Dental implants have become a mainstream treatment approach in daily practice, and because of their high survival rates over time, they have become the preferred treatment option for prosthetic rehabilitation in many situations. Despite the relatively high predictability of implant therapy and high costs to patients, patient perceptions of success and patient‐reported outcome measures have become increasingly significant in implant dentistry. Increasing numbers of publications deal with oral health‐related quality of life and/or patient‐reported outcome measures. The aim of this paper was to provide an overview of the available evidence on oral health‐related quality of life of fully and partially dentate patients rehabilitated with fixed and removable implant‐supported dental prostheses. A comprehensive electronic search was performed on publications in English up to 2021. A selection of standardized questionnaires and scales used for the evaluation of oral health‐related quality of life were analyzed and explained. The analysis encompassed three aspects: a functional evaluation of oral health‐related quality of life, an esthetic assessment of oral health‐related quality of life, and a cost‐related evaluation of oral health‐related quality of life for rehabilitation with dental implants. The data demonstrated that the preoperative expectations of patients markedly affected the outcomes perceived by the patients. As expected, reconstructions supported by implants substantially improved the stability of conventional dentures and allowed improved function and patient satisfaction. However, from a patient's perspective, oral health‐related quality of life was not significantly greater for dental implants compared with conventional tooth‐supported prostheses. The connection of the implants to the prostheses with locators or balls indicated high oral health‐related quality of life. The data also suggest that patient expectation is not a good predictor of treatment outcome. In terms of esthetic outcomes, the data clearly indicate that patients’ perceptions and clinicians' assessments differed, with those of clinicians yielding higher standards. There were no significant differences found between the esthetic oral health‐related quality of life ratings for soft tissue‐level implants compared with those for bone‐level implants. Comparison of all‐ceramic and metal‐ceramic restorations showed no significant differences in patients’ perceptions in terms of esthetic outcomes. Depending on the choice of outcome measure and financial marginal value, supporting a conventional removable partial denture with implants is cost‐effective when the patient is willing to invest more to achieve a higher oral health‐related quality of life. In conclusion, the oral health‐related quality of life of patients rehabilitated with implant‐supported dental prostheses did not show overall superiority over conventional prosthetics. Clinicians' and patients' evaluations, especially of esthetic outcomes, are, in the majority of cases, incongruent. Nevertheless, patient‐reported outcomes are important in the evaluation of function, esthetics, and the cost‐effectiveness of treatment with implant‐supported dental prostheses, and should be taken into consideration in daily practice.

## 1 INTRODUCTION

The models for restoring edentulous patients have changed in recent decades because of the high predictability of oral implants.^1^ Historically, dental implants were installed in fully edentulous patients with the aim of increasing the stability of full denture prostheses.^2^ However, with increasing predictability of implant treatment,^3^ various dental implant‐loading protocols have been proposed, expanding the range of implant rehabilitation protocols for partially edentulous patients.^4^, ^5^ Today, the majority of implants are used to rehabilitate partially edentulous patients, who represent up to 90% of all implant patients.^6^, ^7^ The development of appropriate augmentation techniques and the introduction of novel implant surfaces has resulted in acceptable treatment outcomes, even where esthetics are a priority.^8^ In this respect, surgical and prosthetic procedures have led to improved esthetic outcomes for the teeth to be replaced.^9^, ^10^, ^11^

From the patients’ perspective, an excellent esthetic outcome is perceived as a satisfactory final solution to cope with their dental problems. However, it is well known that biologic complications can arise with dental implant placement, and infections may develop that require a complex, protracted, and costly treatment of peri‐implant infections. Again, the patient is usually unaware of the risks associated with implant placement in terms of biologic complications.

In this context, patients’ perceptions and psychological parameters are becoming more and more significant in evaluating treatment outcomes in implant dentistry.^12^, ^13^ This is reflected in an increasing number of recent publications on patient‐reported outcome measures.^14^ In implant dentistry, patient‐reported outcome measures were proposed at the 8th European Federation of Periodontology Consensus Conference with the aim of focusing on patients’ perspectives and evaluating oral health‐related quality of life.^15^

The aim of this narrative review was to summarize the current evidence on oral health‐related quality of life of fully and partially dentate patients rehabilitated with fixed and removable implant‐supported dental prostheses. Special emphasis was given to oral function, esthetic outcomes, and cost‐related aspects.

## 2 MATERIALS AND METHODS

### 2.1 Methodology

A comprehensive electronic search of the MEDLINE–PubMed database was performed for articles published in English up to 2021, applying the following free text terms: "PROMS" or "Oral health related quality of life" or "patient related outcome measures" or "patient satisfaction" or "esthetics" or "function" or "cost‐effectiveness" or "patient happiness" and "dental implants" or "implant dentistry". Moreover, the reference lists from the retrieved publications were screened to identify any additional relevant studies.

### 2.2 Definition and assessment of oral health‐related quality of life

Oral health‐related quality of life is an established and relevant instrument with which to describe patient satisfaction. It was developed with a psychometric and social survey background and encompasses multiple dimensions of life, ranging from impairment (which is closely linked to clinically defined health status) to social function, and to more global constructs such as “opportunity.” These dimensions have been linked to conceptual models, in which the effects of impairment on disability or reduced opportunity are mediated by intervening with personal and environmental factors.^16^ This makes it difficult to summarize oral health‐related quality of life in a single term. In fact, there is still a lack of consensus on the plethora of terms used in the literature for oral health‐related quality of life.^17^ Moreover, the instruments used to analyze oral health‐related quality of life remain unstandardized and rather heterogenic in nature. Nevertheless, there are many standardized questionnaires and scales employed to assess the impact of dental interventions upon oral health‐related quality of life, and a selection of these questionnaires are listed and explained in Table 1.

**TABLE 1:** Standardized questionnaires to assess OH‐QoL, according to the table of Locker and Allen^17^ and the table of Bennadi and Reddy^23^ with modifications (and with no claim to completeness)

Instrument	Authors	Dimensions measured	No. of items	Answer modality
Social dental scale	Cushing et al (1986)^24^	Chewing, talking, smiling, laughing, pain appearances	14	Yes/no
GOHAI	Atchison and Dolan (1990)^25^	Chewing, eating, social contacts, appearance, pain, worry, self‐consciousness	12	Six categories; “always‐never”
DIP	Strauss and Hunt (1993)^26^	Appearance, eating, speech, confidence, happiness, social life, relationships	25	Three categories; good effect, bad effect, no effect
OHIP	Slade and Spencer (1994)^22^	Function, pain, physical disability, social disability, handicap	49	Five categories; “very often‐never” Short forms: OHIP‐14 = short form with 14 items OHIP‐Edent = short form for edentulous patients
SOHSI	Locker and Miller (1994)^27^	Chewing, speaking, symptoms, eating, communication, social relations	42	Various depending on question format
DIDL	Leao and Sheiham (1996)^28^	Comfort, appearance, pain, daily activities, eating	36	Various depending on question format
OIDP	Adulyanon and Sheiham (1997)^29^	Performance in eating, speaking, oral hygiene, sleeping, appearance emotion	9	Various depending on question format
OH‐QoL measure	Kressin (1997)^30^	Daily activities, social activities, conversation	3	Six categories; “all of time” to “none of the time”
OH‐QoL inventory	Cornell et al (1997)^31^	Oral health, nutrition, self‐related oral health, overall quality of life	56	Part A: 4 categories “not at all” to “a great deal”; Part B: 4 categories “unhappy‐happy
Rand dental health index	Dolan and Gooch (1997)^32^	Pain, worry, conversation	3	Four categories; “not at all” to “a great deal”
Post‐1997 (Conference in Chapel Hill, North Carolina)				
Orthognathic QOL questionnaire	Cunningham et al (2000)^33^	Eating/chewing, pain, social contacts, appearance, self‐consciousness, smiling	22	1 = “it bothers you a little” 4 = “it bothers you a lot” 2 + 3 = “lie between these statements” N/A = “the statement does not apply to you or does not bother you”
COHQoL for children aged 11‐14 y	Jokovic et al (2002)^34^	Symptoms, functional limitations, emotional well‐being, social well‐being	36	Part 1/importance: 4‐point Likert scale (0 = “does not bother me at all”, 4 = “bothers me very much”) Part 2/frequency: 0 = “never”, 1 = “once/twice”, 2 = “sometimes”, 3 = “often”, 4 = “every day/ almost every day” Global ratings for well‐being: 5‐point response 0 = “excellent”/ “not at all”, 5 = “poor”/ “very much”
OH‐quality of life UK	McGrath and Bedi (2003)^35^	Performance in eating, appearance, comfort, speaking, sleeping, social contacts, finances, self‐consciousness	16	Part 1: 3 categories; good, no, or bad effect Part 2: 4 categories: none, little, moderate, great or extreme impact
OIDP for children aged 11‐12 y	Gherunpong Tsakos and Sheiham (2004)^36^	Eating, speaking, cleaning, sleeping, emotion, smiling, studying, social contact	8	0‐3 Likert‐type scales
PPIDAQ	Klages et al (2006)^37^	Social impact, esthetic attitude and dental self‐confidence	23	Likert response: 0 = “never” 1 = “hardly ever” 2 = “occasionally” 3 = “fairly often” 4 = “very often”
SOOQ	Locker et al (2007)^38^	Issues before surgery, issues after surgery, dental esthetics, facial esthetics, emotional and social well‐being	33	4‐point Likert scale Part 1/frequency: “never” to “all the time” Part 2/importance: “not at all” to “very much” Timing: pretreatment, immediate postsurgery (ie, 2‐6 mo) and postsurgery (ie, > 2 y) Short form: 15 items
QoLIP‐10	Preciado et al (2013)^39^	Biopsychosocial, dental‐facial esthetics, and performance	10	Likert‐scale score −2 = “strongly disagree” score −1 = “disagree” score +2 = “indecisive, indifferent, or neutral score 0 = “agree” score +2 = “strongly agree”

Abbreviations: COHQoL, child oral health quality of life questionnaire; DIDL, dental impact on daily living; DIP, dental impact profile; GOHAI, general (geriatric) oral health assessment index; OH, oral health; OH‐QoL, oral health quality of life; OHIP, oral health impact profile; OIDP, oral impacts on daily performances; PIDAQ, psychosocial impact of dental esthetics questionnaire; QOL, quality of life; QoLIP‐10, quality of life with implant prostheses; SOHSI, subjective oral health status indicators; SOOQ, surgical orthodontic outcome questionnaire.

#### 2.2.1 Visual analog scale

The visual analog scale is defined as the distance on a horizontal line between two anchoring points representing the minimum and the maximum perception. The anchoring points are usually 10 cm apart, and the scale on the line is in millimeters or other units. In order to quantify a parameter on a visual analog scale, the evaluator will present a mark on the line. Thus, the distance from the mark to the anchoring point may be calculated.^18^, ^19^ The scale is often used as answer modality in standardized questionnaires (Table 1).

#### 2.2.2 Likert scale

The Likert scale is named after its inventor.^20^ Likert was a psychologist and he used the scale as a technique for evaluation of people's attitudes. The scale contains five points on a horizontal line with a maximal distance between each. Each point is tagged with a descriptor. The patient is summoned to highlight the most accurate description according to their opinion. Today, the scale is widely used in research and it has undergone many adaptations.^18^, ^20^ The scale is employed as a component of several standardized questionnaires (Table 1).

#### 2.2.3 Standardized questionnaires

In the last 3 decades, a variety of standardized questionnaires have been proposed and propagated. The questionnaires usually comprise different areas.

Prior to the conference on oral health‐related quality of life in North Carolina,^16^ there was no consensus regarding how to evaluate oral health‐related quality of life. At that conference, efforts were made to standardize health questionnaires. The questionnaires were analyzed in terms of reliability, validity, and precision. Following the conference, further questionnaires were introduced.^21^ A detailed table of the questionnaires, with descriptions of the dimensions evaluated, as well as the number of questions and answering modalities, is listed in Table 1. One questionnaire in particular is emphasized because it is frequently used in the publications cited in this review: the oral health impact profile.^22^ The oral health impact profile assesses the dimensions of function, pain, physical disability, social disability, and handicap. The patient is asked to answer 49 standardized questions with answering modalities in five categories. A shorter version applying 14 standardized questions has also been validated and propagated.^22^

The methods for the judgment of the esthetics by clinicians are depicted in Table 2.

**TABLE 2:** Methods for judgment of the esthetics by clinicians

Instrument	Authors	Dimensions measured
Papilla height/embrasure fill	Jemt et al (1997)^88^	Five possible scores: 0 no papilla fill; 1 < 50%; 2 > 50%, 3 full papillae; 4 hyperplastic papillae
Level of the mucosal margin	Schropp et al (2008)^89^	In millimeters, comparing the implant site with that of a reference tooth site
Buccal soft tissue dimensions	Thoma et al (2016)^90^	Assessed with endodontic files, standardized stents, ultrasonic devices
Color of the peri‐implant mucosa	Sailer et al (2014)^91^	Spectrophotometers to assess color match between the contralateral, adjacent tooth
Pink esthetic score	Furhauser et al (2005)^80^	Assessed clinically or on photograph including 7 items: mesial papilla, distal papilla, level of soft tissue margin, soft tissue contour, bone deficiencies, soft tissue color, and soft tissue texture A score from 0 to 2 per item. Maximum of 14
Implant crown esthetic index	Meijer et al (2005)^81^	Reconstructive parameters such as dimensions of the crown, position of the incisal edge, etc.
Pink esthetic/white esthetic score	Belser et al (2009)^92^	Combined with reconstruction including 10 items: general tooth form, volume of clinical crown, surface texture, color, translucency, and characterization of the crown
CIS	Hosseini and Gotfredsen (2012)^86^	Six esthetic parameters: (I) crown morphology score, (II) crown color match score, (III) symmetry/harmony score, (IV) mucosal discoloration score, (V) papilla index score, and (VI) mesial papilla index score, distal

Abbreviation: CIS, Copenhagen index score.

## 3 RESULTS

The evaluation of oral health‐related quality of life of patients rehabilitated with dental implants can be summarized in three domains: the aspect of function, esthetics, and cost‐effectiveness. Furthermore, the functional aspect was subdivided into fully edentulous, partially edentulous patients, and the topic of implant‐supported vs tooth‐supported fixed dental prostheses. An overview of the results is summarized in Table 3.

**TABLE 3:** Characteristics of the included studies

Functional evaluation of OH‐QoL and rehabilitation with dental implants						
Ref. number	Authors	Study design	Population	Objective/primary outcome	Assessment tool/ procedure	Results
40	Farzadmoghadam et al (2020)	Retrospective study	102 patients rehabilitated with various implant‐supported reconstructions	Relationship between OH‐QoL and general health‐related quality of life	Subjective assessment: visual analog scale, EuroQol‐5D, and the OHIP‐14 questionnaire	Results indicated an increase in general and oral health‐related quality of life after implant treatment. There was a positive weak relationship between OH‐QoL and general health‐related quality of life
41	Yeung et al (2020)	Non‐randomized controlled trial	104 patients from a private practice were assigned to 3 treatment protocols: the conventional treatment in which implants were inserted after flap elevation without guiding templates; the guided surgery/conventional loading group the guided surgery/immediate loading group	Comparison of 3 prosthetic implant protocols	Subjective assessment: Oral Impacts on Daily Performances and Oral Satisfaction scale	OH‐QoL improved more when the implants were loaded immediately than when the prosthetic rehabilitation was delayed
43	Fonteyne et al (2021)	Prospective study	21 fully edentulous patients received implant‐supported overdentures (2 implants connected with a bar)	Assessment of articulation and its alteration, oro‐myofunctional behavior during 3 stages: pretreatment, during provisional, and after final reconstruction	Assessment: by speech therapists, OH‐QoL, visual analog scale	OH‐QoL increased over treatment process. No impact on speech or oro‐myofunction was found after treatment
44	Dellepiane et al (2020)	Prospective study	25 patients with compromised dentitions were rehabilitated with implant‐supported full arch immediate loading rehabilitation	Assessment of OH‐QoL before, during, and after completion of treatment	OH‐QoL using 4 questionnaires specifically designed for this study to investigate pain, comfort, oral hygiene habits, esthetics, masticatory ability, phonetics, and general satisfaction	96% of the patients did not show esthetic concerns after 4 mo of rehabilitation 92% of the patients did have difficulty eating after 4 mo of rehabilitation OH‐QoL was significantly improved after treatment
45	Zhang et al (2019)	5‐y prospective study	103 geriatric patients with a history of deficient complete dentures	OH‐QoL of patients treated with mandibular two‐implant retained overdentures	Subjective assessment: Own questionnaire (40 items, 4 point rating scale: “not at all” ‐ “extremely”) Objective assessment: Woelfel's index	The support of a full dental prosthesis supplemented by 2 implants improves the retention and stability of the prosthesis significantly Implant‐supported mandibular dentures yielded the best results, as was reflected in reduced functional complaints, complaint frequency and intensity of complaints. Overall patient satisfaction correlated negatively with technical complications
46	Doornewaard et al (2019)	3‐y prospective study, split mouth	Report of two studies. First study: 26 patients received 2 implants Second study: 23 patients received 2 implants	Impact of supported mandibular overdenture on OH‐QoL	Subjective assessment: OHIP‐14 questionnaire	Implant‐supported mandibular overdenture significantly improves the OH‐QoL
47	Yao et al (2018)	Systematic review	‐	Comparing OH‐QoL outcome measures of implant‐supported fixed complete dentures and overdentures	Subjective assessment: OHIP‐14 questionnaire, OHIP‐49 questionnaire, visual analog scale, Likert scale	Fixed and removable implant retained prostheses were rated similarly Only cleansability was rated differently Inconsistent results indicate that the question whether to restore an edentulous patient with either fixed or removable implant prostheses cannot be solely answered by assessing patient‐reported outcomes
48	Coutinho (2021)	5‐y prospective study	30 patients	Impact on OH‐QoL of patients rehabilitated with single‐implant mandibular overdentures	Subjective assessment: OHIP‐Edent	Peri‐implant soft tissue conditions did not change significantly over 5 y. Statistically significant improvement in OH‐QoL was assessed after 5 y compared with baseline Comfort, stability, and ability to masticate was significant increased for single‐implant mandibular overdentures and all evaluation periods
49	Kutkut et al (2018)	Systematic review	‐	Comparing OH‐QoL outcome measures of conventional complete dentures with unsplinted implant‐retained overdentures	Subjective assessment: OHIP‐ questionnaire Visual analog scale Objective assessment: masticatory performance test	Implant‐retained overdentures were associated with significantly better patients' masticatory performance and oral health‐related quality of life. Significantly higher ratings of overall satisfaction, comfort, stability, ability to speak, and ability to chew were associated with patients rehabilitated with mandibular unsplinted implant‐retained overdentures than conventional complete dentures
50	Sivaramakrishnan et al (2017)	Systematic review	‐	Comparing patient satisfaction with mini‐implant vs standard diameter implant overdentures	Meta‐analysis of subjective assessment: Oral health‐related quality of life Visual analog scale OHIP‐14	Mini‐implant‐supported compared with standard diameter implant‐supported overdentures indicated significantly better patient satisfaction levels
51	Sivaramakrishnan et al (2016)	Systematic review	‐	Comparing OH‐QoL outcome measures of implant‐supported mandibular overdentures and conventional dentures	Meta‐analysis of subjective assessment: OHIP‐ questionnaire	Except for physical pain statistically significant better patient satisfaction levels were found for patients treated with implants
52	Allen et al (2006)	Randomized clinical trial (3 mo)	1st group: 62 patients receiving implants 2nd group: 56 patients receiving conventional denture	Comparing OH‐QoL outcome measures of implant‐retained mandibular overdentures and conventional complete dentures	Subjective assessment: OHIP‐ questionnaire Objective assessment Validate denture satisfaction scale	Patients receiving implants showed significantly higher OHIP score changes than patients refusing implant treatments
53	Allen et al (2001)	Prospective study	1st group: 20 patients; edentulous for a mean time of 23.1 y and had worn a mean of 6.7 sets of complete denture prostheses 2nd group: 20 patients; edentulous for a mean time of 19.9 y and had worn a mean number of 4.9 sets of complete denture prostheses 3rd group: 35 patients; edentulous for a mean time of 27.1 y and had received a mean number of 3.4 sets of complete dentures	Comparing OH‐QoL outcome measures of implant‐supported overdentures and conventional dentures	Subjective assessment: OHIP‐ questionnaire and validate denture satisfaction and expectation scale (Likert response format: 1‐5 = “totally satisfied” to “not at all satisfied”)	Patients’ satisfaction improved even in the group of patients who preferred implant‐stabilized prostheses but instead were treated with conventional prostheses. But the extent of patients’ satisfaction was higher with patients who received their desired treatment. Therefore patient expectations did not indicate them to be a good predictor of treatment outcome
54	Heydecke et al (2005)	Randomized clinical trial	102 patients, aged 35‐65 y, had been edentulous for at least 10 y group: patients received mandibular conventional complete dentures group: patients received mandibular overdentures retained by two implants	Assessing the impact of conventional and implant‐supported prostheses on social and sexual activities in edentulous adults	Subjective assessment: OHIP‐ questionnaire and Social Impact Questionnaire	Eating, speaking, kissing, and yawning were significantly improved in the group receiving implant‐supported prostheses Nevertheless there were only weak correlations found between the two sexual activity items (uneasiness when kissing and during sexual relations) and the OHIP scores
55	Zembic et al (2014)	Prospective clinical study	21 patients being edentulous in the maxilla and encountering problems with their existing dentures were included 12 received a new set of conventional dentures; as a consequence of insufficient denture stability (9: 2 women and 7 men), the existing dentures were adjusted by means of relining or rebasing All patients received implant‐supported dentures on two retentive anchors	Comparing OH‐QoL outcome measures of implant‐retained maxillary overdentures and conventional dentures	Subjective assessment: OHIP‐ questionnaire and visual analog scale	Patient satisfaction significantly increased for implant‐supported dentures compared with old dentures in all seven OHIP subgroups, as well as for cleaning ability, general satisfaction, ability to speak, comfort, esthetics, and stability
56	Schuster et al (2020)	Prospective longitudinal clinical study	20 patients rehabilitated with implant‐retained mandibular overdenture after 2 and 3 y	To investigate evolution of masticatory function, OH‐QoL, and prosthetic occurrences of implant‐retained mandibular overdenture wearers according to mandibular bone atrophy over 3 y of usage	Subjective assessment: DIDL questionnaire and OHIP‐14 questionnaire	Masticatory function and OH‐QoL are not related to mandibular bone atrophy until 3 y after implant‐retained mandibular overdenture rehabilitation The DIDL questionnaire showed no significant difference for almost all domains, except for the general performance domain, where a moderate effect was found for the third y
57	Fonteyne et al. (2021)	3‐y prospective study	21 patients receiving implant‐supported overdentures	Impact of four implant‐supported overdenture in the maxilla on OH‐QoL and speech of patients	Subjective assessment: OHIP‐14 questionnaire and visual analog scale	Number of articulation disorders decreased but was not statistically significant Overall satisfaction improved after insertion of connection of implant bar All seven domains improved in OH‐QoL for implant‐supported overdentures compared with conventional dentures
58	Garcia‐Minguillan (2021)	Cross‐sectional study	Test group: 85 endentulous patients 42: conventional denture 43: implant‐retained overdenture Control group: 50 patients with healthy natural dentition	Comparing OH‐QoL of patients with fully dentate subjects and edentulous patients	Subjective assessment: OHIP‐14, OHIP‐20, and Quality of Life with Implant‐Prostheses‐10 questionnaire	Patients with natural dentitions were most critical Patients with implant overdentures showed better OH‐QoL than patients with conventional dentures
59	Kusumoto et al (2020)	Prospective study	72 patients rehabilitated with implant fixed complete dentures or implant overdentures	Association between implant fixed complete dentures and implant overdentures on OH‐QoL	Subjective assessment: OHIP‐49 questionnaire	Except for the perception of masticatory function, both implant‐fixed complete dentures and implant overdentures indicated comparable OH‐QoL
60	Matthys et al (2019)	Comparative clinical cohort	34 patients rehabilitated with balls 56 patients rehabilitated with locators	To assess 5 y of clinical implant outcome, prosthetic maintenance, cost, and OH‐QoL of two cohorts receiving 2 implant over dentures on ball or stud abutments	Subjective assessment: OHIP‐14 questionnaire	Balls and locators yield stable 5‐y implant outcome and improved OH‐QoL. OHIP‐14 declined from 18.1 to 2.7 for both attachment modalities Locators required more maintenance and resulted in a lower retention. Maintenance costs are minimal but may affect OH‐QoL
61	Brandt (2021)	Retrospective study	122 patients	Comparing OH‐QoL of patients receiving ball vs Locator attachments for implant‐retained overdentures	Subjective assessment: OHIP‐14	Patients receiving Locator attachments indicated significant better OH‐QoL compared with patients receiving balls attachment
62	Negoro (2021)	Prospective study	30 patients with Kennedy class I or II and distal extension defects of 3 or more teeth	Comparing OH‐QoL of patients with conventional removable partial dentures, (short) implant‐assisted removable partial dentures, and with or without magnetic attachments	Subjective assessment: OHIP‐49	The OH‐QoL was significantly increased for patients receiving implant‐assisted removable partial dentures with magnetic attachments compared with rehabilitation without magnetic attachments
63	Zhou	Up to 5‐y retrospective study	48 patients treated with implant‐retained mandibular overdentures Group A: 26 patients treated with bar attachments Group B: 22 patients treated with magnetic attachments	Comparing OH‐QoL of patients receiving ball vs magnetic attachments for implant‐retained mandibular overdentures	Subjective assessment: visual analog scale	Peri‐implant probing depth and plaque index were significantly better for the magnetic attachment group compared with the bar attachment group OH‐QoL was not statistically significantly different between both groups Nevertheless, patients treated with bars had significantly more difficulties to clean their reconstructions than patients treated with magnetic attachments
64	Gündoğar (2021)	Cross‐sectional study	109 geriatric patients	Impact of peri‐implant disease on OH‐QoL in a geriatric population	Subjective assessment: OHIP‐14	Prevalence of peri‐implantitis was 30%. Prevalence of peri‐implant mucositis was 24% Statistical analysis failed to reveal any significance between patients with peri‐implantitis or peri‐implant mucositis. Plaque index and gingival index were statistically significantly correlated with total OHIP‐14 score
65	Thomason et al (2007)	Systematic review	‐	How do reconstructions affect patient‐reported outcomes of conventional dentures vs implant‐supported overdentures	QoL, OH‐QoL, patient satisfaction (with a range of parameters)	The overall rating for OH‐QoL of patients receiving implant‐supported overdentures was not significantly better than for conventional dentures
66	Tsakos et al (2006)	National Diet and Nutrition Survey	Sample of the National Diet and Nutrition Survey (people aged ± 65 y) 407 dentate and 346 edentate participants	OH‐QoL of life correlations in a national geriatric sample	Subjective assessment: OIDP ‐ questionnaire	Patients with > 8 occluding pairs of teeth were 2.66 times, and those with up to 2 anterior occluding pairs, were 3.00 times less likely to report oral impacts Edentate participants with inadequate denture adaptation and subjects with inadequate denture retention were more likely to report oral impacts than the remaining edentate patients In each case OH‐QoL is significantly related to the number of occluding pairs of natural teeth among the dentate and denture quality among the edentate
67	Steele et al (2004)	National sample	UK, 1998: Adult Dental Health Survey Australia, 1999: National Dental Telephone Interview Survey	Impact of tooth loss on OH‐QoL	Subjective assessment: OHIP‐14	Patients with average number of teeth showed significantly better scores than all other groups with less teeth Important variables influencing OH‐QoL are age, number of teeth, and cultural background Australian‐ and British‐born groups showed better overall scores compared with first‐generation immigrants from elsewhere
68	Wong et al (2005)	Retrospective study	233 patients; 60‐80 y old	Impact of tooth loss on emotion/OH‐QoL for edentulous and partially dentate patients	Subjective assessment: General Oral Health Assessment Index	22% of patients had difficulty in accepting tooth loss Edentulous patients were significantly more satisfied with their prostheses compared with partially dentate patients
69	Kurosaki et al (2020)	Retrospective study	105 partially edentulous patients received 1 out of 3 prosthetic treatments and were followed 6 y	Long‐term performance of 3 prostheses: implant‐supported fixed denture, FPD, and removable partial denture in terms of survival and OH‐QoL	Subjective assessment: Oral Health‐related Quality of Life, psychological health‐related quality of life, a previously validated questionnaire, which was developed based on the OHIP	Implant‐supported fixed denture, FPD, and removable partial did not yield statistical significantly differences in terms of OH‐QoL
70	Dong et al (2019)	Prospective study	373 patients	OH‐QoL outcome measures of patients rehabilitated with single implants	Subjective assessment: OHIP‐14, Oral implant profile questionnaire, visual analog scales, open‐ended question: “What was the most concerning aspect that affected your satisfaction in the implant treatment?”	Patients treated with bone augmentation and those with a longer period of tooth loss are significantly less satisfied Patients are most concerned about survival time success rate and survival time of implants
71	AlZarea et al (2017)	Prospective study	79 partially edentulous patients	OH‐QoL of partially edentulous patients rehabilitated with implant‐supported single crowns or FPDs (pre‐and post‐treatment)	Subjective assessment: OHIP‐49 (pre‐and post‐treatment)	From baseline to 1st y of observation a significant increase of patients’ OH‐QoL in terms of functional limitation, physical pain, psychological discomfort, physical disability, psychological disability, and social disability but not handicap was found From baseline to 2nd and 3rd y all variables also significantly indicated an increase of OH‐QoL There were no significant differences between gender
72	Gerritsen et al (2010)	Systematic review	‐	Impact of tooth loss on OH‐QoL	Subjective assessment: OHIP‐49, OHIP‐14, GOHAI, OIDP, ad hoc satisfaction questionnaires	The results indicated that not only number of tooth loss, but location and distribution of missing teeth, affect the reduction of OH‐QoL. Furthermore, the extent and severity of impairment seems to be context‐dependent (eg, cultural background)
73	AlZarea et al (2016)	Retrospective study	92 patients	OH‐QoL of patients rehabilitated with dental implants	Subjective assessment: OHIP‐14	Results from the OHIP‐14 questionnaire revealed that patients with dental implants were satisfied with their OH‐QoL
74	Sargozaie et al (2017)	Cross‐sectional study	73 patients	OH‐QoL of patients rehabilitated with dental implants (pre‐and post‐treatment)	Subjective assessment: OIDP	Before surgery, the most common problems reported were eating, smiling, laughing, and embarrassment. After surgery, eating, speaking clearly, clean teeth or dentures, light physical activities, smiling, laughing, showing teeth without discomfort and embarrassment, emotional conditions, enjoying communication with others, and job‐related activities significantly increased OH‐QoL But after surgery the amount of sleep and resting did not improve. After implantation, the place of residence, education, and gender did not show a significant association with OH‐QoL
75	Reissmann et al (2017)	Systematic review	At least 50 patients	OH‐QoL of patients rehabilitated with implant‐supported prosthesis	Subjective assessment: OHIP‐ questionnaire, Geriatric Oral Health Assessment Index, UK oral health‐related quality of life measure, and DIDL	For partially dentate patients, implant‐supported FDPs did not show superiority over conventional fixed dental prostheses Implant‐supported FDPs indicated moderate superiority over conventional removable dental prostheses For edentulous patients that are, at baseline, highly impaired and requested implant treatment, improvements of OH‐QoL was superior for implant‐supported dentures compared to conventional dentures
76	Cadel‐Marti et al (2015)	Retrospective study	57 patients with severely atrophic maxillae	Comparing OH‐QoL of patients treated with partial positioned implants vs conventional implants supporting fixed full‐arch prostheses	Subjective assessment: OHIP‐14 questionnaire and visual analog scale	Patients treated with partial positioned implants (more coverage of palate) vs conventional implants supporting fixed full‐arch prostheses did not show reduction of OH‐QoL
77	Torres et al (2011)	Prospective study	50 patients with implant‐supported mandibular overdentures 50 patients with conventional mandibular dentures	Impact of personality traits on OH‐QoL of patients treated with conventional mandibular dentures and implant‐supported overdentures	Subjective assessment: OHIP‐14 questionnaire and Neuroticism Extraversion Openness Five‐Factors Inventory (five personality domains)	Patients with conventional mandibular dentures reported higher levels of impact on OH‐QoL compared with patients with implant‐supported mandibular overdentures OH‐QoL is significant associated with personality traits (mainly neuroticism) related to implant‐supported or conventional removable complete dentures

Esthetic assessment of OH‐QoL in conjunction with oral rehabilitation with dental implants						
Ref. number	Authors	Study design	Population/ examiner	Objective	Assessment Tool/ Procedure	Results
78	Yu et al (2013)	6‐month prospective study	238 patients	Impact of missing anterior teeth rehabilitated with implants on OH‐QoL	Subjective assessment: OHIP‐14	After crown restoration, OH‐QoL of patients increased statistically significantly compared with the situation before implantation
79	Wang et al (2021)	Cross‐sectional survey	95 patients receiving fixed implant‐supported restorations	Assessment of patients’ satisfaction regarding function (phonetics, chewing comfort, stability, cleansability) and esthetics in a peridontal practice 10 y after implant placement	Subjective assessment: visual analog scale, OHIP, and Psychosocial Impact of Dental Aesthetics Questionnaire	Mean visual analog scale score, mean OHIP, and mean Psychosocial Impact of Dental Aesthetics Questionnaire scores were 93%, 11.3, and 20.5, respectively. Therefore patients showed high satisfaction with their restorations
82	Vaidya et al (2015)	Evaluation of photographs	Evaluation of 20 intra‐oral photographs: 14 examiners (2 orthodontists, 2 prosthodontists, 2 oral surgeons, 2 periodontists, 2 dental technicians, 2 dental assistants, and 2 postgraduate students in Implant Dentistry)	Impact of clinicians with diverse dental backgrounds on the evaluation of maxillary implant‐supported single‐tooth replacements	Objective assessment: Pink Esthetic Score/White Esthetic Score and the modified Implant Crown Esthetic Index	Pink Esthetic Score/White Esthetic Score and the modified Implant Crown Esthetic Index showed reliable estimates of esthetic outcomes The degree of specialization of the clinician affect the esthetic evaluation for both indices: Pink Esthetic Score/White Esthetic Score and the modified Implant Crown Esthetic Index. Prosthodontics were most critical. DAs and periodontists provided favorable ratings compared with other specialties
83	Chang et al (1999)	Evaluation of photographs	Intra‐oral and extraoral photographs were taken from 29 patients with 41 single implant‐supported crowns in the maxillary anterior region and were included 5 prosthodontists evaluated the photographs	Are prosthodontics’ and patients’ evaluations of esthetic outcomes of implant‐supported single‐tooth replacements different?	Subjective assessment: visual analog scale	Subjective assessment was found to be higher for all variables compared with the clinician’s evaluation Factors considered important from the clinician’s view may not be decisively important for patients’ satisfaction
84	Esposito et al (2009)	Evaluation of photographs	30 patients evaluated their own results 10 clinicians evaluated all 30 patients’ results	Are prosthodontics’ and patients’ evaluations of esthetic outcomes of implant‐supported single‐tooth replacements different?	Subjective assessment: visual analog scale Evaluation of intra‐oral and extraoral photographs	Clinicians’ responses were less in agreement than patients’ responses Agreement between patients’ and clinicians’ responses was poor
85	Wittneben et al (2018)	Systematic review	816 implant‐supported reconstructions were analyzed by patients	Patient‐reported outcome with focus on esthetics	Subjective assessment: visual analog scale	Visual analog scale evaluation (rated by patients) for esthetic outcome of fixed dental prostheses was high For the implant‐supported FDPs and the surrounding mucosa, the visual analog scale evaluation from patients was also high No effect on patients’ satisfaction of the definitive implant‐supported fixed dental prostheses with the following parameters: individual restorative materials, implant neck design (ie, tissue or bone level type implants), and the use of a fixed provisional
87	Hosseini et al (2013)	3‐y prospective study	Supported, single tooth restorations	Comparing patient‐reported outcome of all‐ceramic vs metal‐ceramic crowns of implant reconstructions.	Subjective assessment: OHIP‐49 Objective assessment: Copenhagen Index Score^67^	The 3‐y survival rate for implants was 100% and for abutments was 97% Significantly more marginal bone loss was found for gold‐alloy abutments compared with zirconia abutments The most frequent technical complication was loss of retention, which was only found in metal‐ceramic crowns All‐ceramic restorations showed more frequently biologic complications than metal‐ceramic crowns. Marginal adaptations of all‐ceramic crowns were significantly less optimal than metal‐ceramic crowns. Significant better color match for all‐ceramic compared with metal‐ceramic restorations was reported from professionals. No significant discrepancies in patients’ satisfaction for esthetic outcome was found for patients rehabilitated with all‐ceramic vs metal‐ceramic restorations

Cost‐related evaluation of OH‐QoL and rehabilitation with dental implants						
Ref number	Author	Study design	Population	Objective	Assessment tool/Procedure	Results
93	MacEntee et al (1998)	Framework for analysis and preliminary outcomes	‐	Economic aspects of complete dentures and implant‐related reconstruction	Analysis of measurement, distribution, impact, and management: four foundations for the framework	There are physiologic and psychosocial costs and benefits to both the conventional denture and the implant prosthesis, which indicates that neither method is distinctly superior. The physiologic costs are low and the psychosocial costs are similar for both treatments, whereas the direct financial costs associated with the implant prosthesis are substantially higher
94	Bragger et al (2005)	Retrospective study	37 received 41 conventional three‐unit FPDs 52 patients received 59 single crowns on implants (I)	Economic aspects of single‐tooth replacement	Comparison of the two treatments in terms of treatment time, number of visits, monetary and opportunity costs, and comparison	Treatment time was similar, I. required more visits. Total costs were lower for I. Costs for treatment of complications were similar for both groups. Overall, for a short period of time, implant reconstruction showed a more favorable cost/effectiveness ratio
96	Hettiarachchi et al (2018)	Systematic review	‐	The cost‐effectiveness of oral health interventions: a systematic review of cost‐utility analyses	Assessment of cost‐utility analysis in oral health interventions	From 2011 to 2016, the cost‐utility analysis of oral health interventions increased Consolidated Health Economic Evaluation Reporting Standards were used to evaluate the cost‐effectiveness
97	Jensen et al (2017)	Prospective study	30 patients with full upper dentures and complaints regarding their bilateral mandibular free‐ending removable partial dentures were included All patients received 4 implants in the premolar region and new removable partial dentures	Cost‐effectiveness of implant‐supported mandibular removable partial dentures	Cost‐effectiveness analysis comparing conventional removable partial dentures with implant‐supported removable partial dentures in patients with edentulous maxilla Treatment effect was expressed by the Dutch Oral Health Impact Profile Questionnaire OHIP‐NL49	Depending on the choice of outcome measure and monetary threshold, supporting a removable partial denture with implants is cost‐effective when payers are willing to pay > 80 Euros per OHIP point gained
98	Palmqvist et al (2004)	Prospective randomized clinical study	11 patients received fixed prosthesis on 3 implants 6 patients received overdentures on 3 implants and a Dolder bar	Comparing prosthodontic production time and costs in implant‐supported fixed prostheses vs overdentures in the edentulous mandible	Assessment of laboratory and clinical working hours along with cost evaluation	Mean clinical working hours were 3.1 in the fixed prosthesis group and 4.1 in the overdenture group. Mean laboratory working h were 12.5 in the fixed prosthesis group and 7.7 in the overdenture group. Total laboratory costs were on average about 1700 US dollars for the fixed prosthesis and 1350 US dollars for the overdenture
99	Ravida et al (2018)	Retrospective study	45 patients test group: 149 implants control group: 111 implants minimum follow‐up: 5 y mean follow‐up: 9.6 y	Clinical outcomes and cost effectiveness of computer‐guided vs conventional implant‐retained hybrid prostheses	Analysis of patient‐focused costs in terms of necessary costs of diagnostic, therapeutic, and follow‐up procedures	Biologic and technical complications did not show significant differences between groups Incidence of implant loss was less observed in test group The initial cost for the guided implant placement group was significantly higher For the prosthetic complication and total cost, no significant difference was detected between both groups
100	Ravida et al (2013)	Retrospective study	145 patients: 40 nonsplinted crowns 52 splinted crowns 53 implant‐supported bridge 382 bone‐level implants: 120 nonsplinted crowns 106 implant‐supported bridge 156 splinted crowns mean follow‐up: 76.2 mo	Comparing the cost‐effectiveness of three different types of implant‐supported FDPs: rehabiliation of 3‐unit edentulous area	Cost‐effectiveness analysis comparing nonsplinted crown, splinted crowns, implant‐supported bridge	Implant survival rates were 92.5% for nonsplinted crowns, 100% for implant‐supported bridges, and 88.5% for splinted crowns Implant survival rates were significantly different between the implant‐supported bridge and splinted crowns groups Nonsplinted crowns and splinted crowns showed higher total costs compared with the implant‐supported bridge group A 3‐unit implant‐supported bridge on 2 implants showed better results in the long term compared with nonsplinted crowns and splinted crown solutions on implants

Abbreviations: DAs, Dental Assistants; DIDL, dental impact on daily living; FPDs, fixed partial dentures; GOHAI, Geriatric oral health assessment index; OHIP, oral health impact profile; OH‐QoL, oral health‐related quality of life; OIDP, oral impacts on daily performances.

### 3.1 Functional evaluation of oral health‐related quality of life of patients rehabilitated with dental implants

The functional aspect of oral health‐related quality of life is not only important in the field of dentistry. Oral health‐related quality of life also affects the overall well‐being of the individual. In other words, oral health‐related quality of life correlates to general health‐related quality of life.^41^ In terms of rehabilitation with dental implants and the different surgical protocols, it appears that immediate loading protocols achieved the highest patient satisfaction.^42^

#### 3.1.1 Fully edentulous patients

The majority of studies dealing with implant placement and oral health‐related quality of life has been performed in edentulous patients. Edentulism may be associated with functional impairment, which includes chewing ability, bite force, swallowing mechanism, differences in salivary flow, phonetics, and oral sensory function in general. Moreover, cleansability, as well as social behavior, are included. Ill‐fitting and unstable prostheses are a particular source of distress and reduced self‐esteem. Therefore, implant placement in edentulous patients severely impacts on their functional well‐being.^42^

Fonteyne et al^43^ evaluated patients receiving new fully removable dentures during different stages of treatment. The assessments were conducted by speech therapists. Interestingly, despite existing articulation and oro‐myofunctional impairments following treatment, patients were very satisfied with their oral health‐related quality of life and their speech.^43^ Similarly, Dellepiane et al^44^ assessed 25 patients at different stages (ie, before treatment, during the healing phase, and after the final reconstruction). At the final evaluation, 4 months after rehabilitation, 92% of patients did not indicate any difficulty in eating. Overall, oral health‐related quality of life revealed a significant improvement in terms of quality of life, and the patients only reported phonetic impairment in the immediate aftermath of surgery.^44^ Two prospective studies indicated that implant‐supported prostheses improved the oral health‐related quality of life significantly.^45^, ^46^ It has been shown that the support of a full dental prosthesis supplemented by two implants improves the retention and stability of the prosthesis significantly.^42^ A total of 45 edentulous patients receiving implant‐supported mandibular overdentures reported a strong improvement in oral health‐related quality of life in the first 3 years.^43^ Similar results were reported in another prospective study including 67 patients over 5 years. New complete mandibular dentures led to significant improvements in patient‐reported outcome measures. Implant‐supported mandibular dentures yielded the best results, as reflected by reduced functional complaints, complaint frequency, and intensity of complaints. Overall patient satisfaction correlated negatively with technical complications.^47^ An International Team for Implantology consensus report evaluated patient‐reported outcome measures of fixed and removable implant‐retained prostheses in edentulous patients. In the 13 studies included, fixed and removable implant‐retained prostheses were rated similarly. Only cleansability was rated differently. Consequently, whether to restore an edentulous patient with either fixed or removable implant prostheses cannot be solely answered by assessing the oral health‐related quality of life.^47^ Oral rehabilitation of edentulous patients with both implant‐supported fixed and removable prostheses yielded similar patient‐reported outcome measures, as reported in a systematic review.^47^ However, fixed prostheses displayed a trend for higher patient acceptance than removable prostheses. Overall, there is a large body of evidence that implant‐supported overdentures, especially in the edentulous mandible, lead to improved satisfaction in terms of oral health‐related quality of life compared with conventional prostheses.^14^ A 5‐year prospective study including 30 patients rehabilitated with single‐implant mandibular overdentures revealed significant increases in comfort, stability, and the ability to masticate for all evaluation periods.^48^ This is in agreement with a systematic review comparing conventional complete dentures and implant‐retained overdentures.^49^ Implant‐retained overdentures received higher ratings in terms of overall satisfaction, comfort, stability, psychological comfort, chewing function, and ability to speak.^49^, ^50^, ^51^ In a randomized trial including 118 patients, comfort, stability, and retention, as well as chewing function, were reported as being superior for implant‐supported overdentures compared with conventional dentures.^52^ However, where speech or cleaning ability were concerned, patients reported similar results.^53^, ^54^ Furthermore, patients reported an improvement in social as well as in couple activities.^54^ Concerning functional aspects, cleaning, speaking, and pronunciation, overall comfort and stability were evaluated as superior for maxillary implant‐supported overdentures compared with conventional dental prostheses.^55^

The question arises as to whether bone atrophy has an impact on the oral health‐related quality of life of patients treated with implant‐retained overdentures, foremost in the mandibular region. A 3‐year prospective study compared patients treated with implant‐retained mandibular overdentures with and without atrophic mandibles and, interestingly, did not reveal any statistical significant differences.^56^ More recent studies support the finding that retention and therefore better stability of prostheses appears to be an important factor for patients’ oral health‐related quality of life.^57^, ^58^ A 3‐year prospective study assessed the impact of four implant‐supported overdentures in the maxilla in terms of oral health‐related quality of life and speech. Twenty‐one patients were examined preoperatively and following the connection to an implant bar. The design of the conventional denture before surgery was with palatal coverage. The implant‐supported overdenture after treatment was designed without palatal coverage. All seven domains improved oral health‐related quality of life for implant‐supported overdentures compared to conventional dentures.^58^ By contrast, a recent prospective study failed to show significant differences between the aforementioned treatment modalities.^59^ Nevertheless, implant retention of overdentures does appear to be an important driver of patient satisfaction. The connections between implants and the denture are diverse. The different attachment modalities (ie, magnetic, locator, or ball attachments) were assessed in recent studies.^60^, ^61^, ^62^ The results indicated stable 5‐year outcomes and improved oral health‐related quality of life for both locators and balls. Although locators required more maintenance and resulted in lower retention in one study,^60^ another retrospective study (including a total of 122 patients) reported higher patient satisfaction for locators compared with ball attachments.^61^ Oral health‐related quality of life was significantly increased for patients receiving implant‐assisted removable partial dentures with magnetic attachments compared with rehabilitation without magnetic attachments.^62^ However, no difference in terms of oral health‐related quality of life was discerned between bar attachment and magnetic attachment.^63^

With rehabilitations using implants, there is always a risk of peri‐implant diseases. An interesting cross‐sectional study, including 109 geriatric patients, revealed a statistically significant correlation between total oral health impact profile‐14 score, plaque index, and gingival index. These results suggest that peri‐implant health also affects patient satisfaction.^64^Another aspect to be taken into consideration is the effect of preoperative patient expectations on oral health‐related quality of life. This question was addressed in a randomized controlled study including three experimental groups of edentulous patients requesting replacement of their conventional complete dentures.^53^ The first group of 20 patients had been edentulous for a mean time of 23.1 (range 3‐40) years, had worn a mean of 6.7 (3‐12) sets of complete dentures, and requested implants to stabilize their conventional prosthesis in the mandible. They received mandibular implant‐supported fixed partial dentures. In the second group, 20 patients requested an implant‐stabilized prosthesis but did not receive this treatment. Instead they received conventional complete dentures. They had been edentulous for a mean time of 19.9 (2‐35) years and had worn a mean number of 4.9 (1‐12) sets of complete dentures. In the third group, 35 patients requested and received conventional complete dentures. They were treated according to their wishes. In this group the subjects had been edentulous for a mean time of 27.1 (3‐40) years and had received a mean number of 3.4 (1‐12) sets of complete dentures. The results from the oral health impact profile questionnaire indicated that patient satisfaction improved, even in the group of patients who preferred implant‐stabilized prostheses but were instead treated with conventional prostheses. But the extent of patient satisfaction was higher in patients who received their desired treatment. It was concluded that patient expectation is not a good predictor of treatment outcome.^53^

In conclusion, there is increasing evidence that the use of dental implants to support either fixed or removable dental prostheses in fully edentulous patients significantly improves overall patient satisfaction when focusing on functional ability, especially in the lower jaw.^65^

#### 3.1.2 Partially edentulous patients

Replacing missing single teeth with dental implants has become a routine procedure for the rehabilitation of partially edentulous patients, although the lack of a periodontal ligament may have an impact on chewing sensation. The number of missing teeth is reflected in the oral health‐related quality of life^66^, ^67^ assessment, demonstrating that tooth loss per se affects patients psychologically. Recently, it was reported that tooth loss had a negative impact on eating in public and forming close interhuman relationships.^68^

Kurosaki et al^69^ compared the long‐term performance of three different prosthetic reconstruction types—implant‐supported fixed denture, fixed partial denture, and removable partial denture—in terms of prosthetic survival and oral health‐related quality of life. Concerning survival, the 6‐year cumulative survival rates of the implant‐supported fixed dentures, fixed dentures, and removable partial dentures were 94.7%, 77.4%, and 33.3%, respectively. The oral health‐related quality of life scores for the implant‐supported fixed denture group immediately after treatment and 6 years after treatment were significantly higher than those observed before treatment. However, there was no improvement in the oral health‐related quality of life scores in the fixed partial denture or removable partial denture groups compared with before treatment.^69^

According to a questionnaire‐based survey, only 15.3% of patients receiving single implants considered masticatory function as their major concern. A total of 8.6% were most worried about food impaction and another 4.5% about pronunciation, occlusion, and swallowing.^70^ Functional limitations, along with psychological discomfort, significantly decreased in patients who had received implant‐supported single crowns or fixed partial dentures over a period of 3 years following implant installment.^71^

A systematic review with a meta‐analysis, including different questionnaires such as oral health impact profile‐49, oral health impact profile‐14, geriatric oral health assessment index, oral impacts on daily performances, and ad hoc satisfaction, indicated that not only the number of teeth lost, but also the location and distribution of missing teeth, affect the reduction in oral health‐related quality of life. Furthermore, the extent and severity of impairment appears to be context‐dependent (eg, cultural background).^72^

An evaluation of implants and their contralateral teeth clinically, alongside patient satisfaction by oral health impact profile‐14, confirmed that patients with implants were highly satisfied with their oral health‐related quality of life. The majority (72.8%) felt that they were never limited in function. Moreover, they indicated satisfaction with their dietary consumption (69.5%). Nearly half of the patients (48.9%) had encountered phonetic problems pretherapy and had become more self‐confident through implant treatment.^73^

Comparing the oral health‐related quality of life pre‐ and post‐implant placement revealed that, prior to surgery, patients reported functional problems, specifically eating (78%), but also speaking and smiling, which when considered together were a cause of general embarrassment (53%).^74^ After implant placement, the oral health‐related quality of life changed in many aspects. Besides those functions directly related to tooth replacement, such as eating, speaking, or oral sensory function, oral health‐related quality of life increased in general terms. Going out or meeting others, communication, smiling, and showing teeth without discomfort became natural and enjoyable. Interestingly, patients reported that becoming upset, in general, as well as job‐related activities, significantly improved after implant placement.^74^

#### 3.1.3 Implant‐supported vs tooth‐supported fixed dental prostheses

A recent review suggested that in partially dentate patients there was insufficient evidence that implant‐supported fixed dental prostheses yielded better oral health‐related quality of life scores than tooth‐supported fixed dental prostheses.^75^ In partially dentate patients, the consensus of oral health‐related quality of life studies is that treatment with implant‐supported fixed dental prostheses improved oral health‐related quality of life. However, all of these studies need to be interpreted with caution. First, it is clear that all these patients had an edentulous gap or a provisional prosthesis before treatment. It is well known that fabrication of new definitive prostheses positively influences oral health‐related quality of life.^76^ Thus, it remains plausible that the prosthetic replacement rather than the implants per se were responsible for the improved oral health‐related quality of life. Second, patients restored with implants usually have higher levels of education and income, which may affect their satisfaction scores.^77^

To date there is limited evidence for partially dentate patients that implant‐supported fixed dental prostheses are superior in terms of patient perception than conventional fixed dental prostheses.

### 3.2 Esthetic assessment of oral health‐related quality of life in patients rehabilitated with dental implants

Loss of teeth, particularly in the anterior region, is associated with esthetic impairment and reductions in oral health‐related quality of life. In fact, a prospective study with 238 participants investigated patients with loss of anterior teeth and their satisfaction before implantation and following crown delivery. The results showed that oral health‐related quality of life, assessed with the oral health impact profile‐14 questionnaire, increased significantly after crown insertion,^78^ and the effect appeared to be longlasting.^79^ A cross‐sectional study including a total of 95 patients revealed high satisfaction in terms of esthetics and function with implant‐supported restorations, even 10 years after they had received their implants.^79^

For many years, the evaluation of esthetic outcomes of care was obscure and not standardized in the dental literature. However, in 2005, two esthetic assessment methods were proposed and validated, namely, the pink esthetic score^80^ and the implant crown esthetic index.^81^ Both systems were successfully used in subsequent reports on esthetic outcomes in implant dentistry (Table 2). It has to be kept in mind, however, that both systems require the ability to compare the implant reconstruction with a contralateral or control tooth. Nevertheless, the index systems helped clinicians to objectively assess the esthetic aspects of newly placed and reconstructed implants. However, esthetic assessment by means of the index system described is significantly affected by the paradigms of the respective specialties.^82^ In a validation study, prosthodontists were the most critical evaluators and yielded the lowest mean rank scores regardless of the index, while dental assistants and periodontists had significantly better ratings than other specialties.^82^

Hence, it has to be realized that esthetic scores are dependent on the professional experience of the examiners, irrespective of the esthetic index system utilized.^82^ In that respect, a recent review^83^ applied objective and subjective criteria for clinicians and patients to evaluate esthetic outcomes. In that review, the oral health impact profile and oral health‐related quality of life questionnaires were used for esthetic evaluation (Table 2). These standardized and validated questionnaires allowed comparisons. A comparison of the objective and subjective assessments yielded a discrepancy between subjective patient‐related criteria and objective prosthodontist‐related evaluations.

An important aspect to be mentioned is that professionals were more critical than patients when subjective patient‐evaluation was used.^83^ In one study, five prosthodontists were asked to evaluate the esthetic outcome of single implant‐supported crowns based on intra‐oral and extraoral photographs.^83^ A total of 41 implants were placed in the maxillary anterior region of 29 patients. In 89% of cases, the clinicians correctly located the single implant‐supported crown. The form of the crown and surrounding soft tissue were the most important parameters for the clinicians’ satisfaction. However, regression analysis failed to reveal any statistically significant agreement when the patient's view was taken into consideration, as patient satisfaction was higher for all variables compared with clinicians’ satisfaction.^83^

A further study on subjective and objective evaluations of esthetic outcomes in implant dentistry involved 30 patients treated with dental implants. Preoperative and postoperative images were graded using a visual analog scale. At the same time the images were shown to 10 independent clinicians using the same visual analog scale. The same outcome was reported and agreement between patients' and clinicians' evaluations was poor.^84^

A systematic review summarizing the existing evidence on esthetic oral health‐related quality of life of implant‐ and tooth‐supported fixed dental prostheses yielded no significant differences between the ratings for soft tissue‐level implants compared with those for bone‐level implants. The review encompassed 16 publications with a total of 19 relevant study cohorts, covering 816 implant‐supported reconstructions to be analyzed by patients. Despite the high heterogeneity among studies, the authors concluded that the esthetics of implant‐supported fixed dental prostheses are more highly rated by patients than by professionals.^85^

All‐ceramic and metal‐ceramic restorations were compared in a prospective study. A total of 59 patients with tooth agenesis were treated and followed up for 3 years. Finally, a total of 98 implant‐supported single unit crowns were evaluated. Materials used for crowns were either all‐ceramic or metal‐ceramic. Zirconia, titanium, and gold alloys were used for abutments, which retained these crowns. Patient‐reported and professionally reported esthetic outcomes were assessed with the oral health impact profile‐49 questionnaire and the Copenhagen index score, respectively.^86^ The professionals reported significantly superior color match of all‐ceramic over metal‐ceramic crowns. Patient reports for esthetic outcomes did not show a significant discrepancy between restoration materials after 3 years.^87^

It is important to understand that esthetic outcomes should be evaluated separately for partially and fully edentulous patients. It is clear that patients in need of a single unit crown in the frontal region of the maxilla may have higher expectations than fully edentulous patients in need of implant‐supported overdentures. Obviously, patient priorities will be driven by individual differences in their perceived need. In addition, the patient is confronted with proportionally higher costs for a single crown compared with those for an implant‐supported overdenture. It is, therefore, important to understand patient satisfaction scores in conjunction with cost‐effectiveness, which is analyzed in the following section.

### 3.3 Cost‐related evaluation by patients rehabilitated with dental implants

Introducing oral implants as a treatment for partially edentulous patients to improve their quality of oral health was usually accompanied by an increased cost compared with traditional removable prosthetic treatment.^93^, ^94^ In relation to economic factors encountered with a specific treatment, and in comparison with the benefits of such treatments, cost‐utility analyses have been performed. Cost‐utility analysis is a specific model, in which costs are expressed in monitory units, and outcomes of the treatment are assessed as a combination of health improvements in terms of oral health‐related quality of life.^95^ A systematic review indicates the increasing interest of the profession in this topic. It was reported that 60% of the studies in the final analysis (n = 14) had been published during 2011‐2016.^96^ Among several different dental conditions analyzed (ie, oral cancer, dental prostheses, caries prevention, periodontitis), oral prosthetic rehabilitation was investigated in 26% of cases (six publications), highlighting its relevance in terms of cost‐related parameters.^96^ Costs related to implant‐supported dental prostheses have been a focus of discussion for many years. When conventional dentures were compared with fixed prostheses on five implants, a 17‐fold higher cost was found for the fixed overdentures. When fixed dentures on five implants were compared with two implant‐supported removable overdentures, the costs doubled for the fixed solution.^74^ A recent study conducted on 30 partially edentulous patients rehabilitated with conventional removable partial dentures and implant‐supported removable partial dentures included the oral health‐related quality of life aspect in the cost analysis. When a patient was willing to pay > €80 per oral health impact profile point gained, then the implant‐supported removable partial dentures were cost‐effective; however, it also depended on the chosen outcome measure and the financial marginal value.^97^ A randomized prospective study aimed to evaluate the cost‐efficacy of implant‐supported fixed prostheses and conventional dentures retained by a Dolder bar system in the edentulous mandible. Several parameters were considered, namely, treatment results, clinical working hours, laboratory working hours, and laboratory costs (including materials). It was concluded that no significant differences in costs were found between both groups.^98^ A comparison of the cost‐effectiveness of different fixed treatment modalities to rehabilitate posterior partial edentulous spaces with computer‐guided implant placement has recently been performed. However, whether or not computer‐guided implant placement will result in higher oral health‐related quality of life standards remains controversial, even although there were significant differences in favor of the nonguided implant placement group for the initial costs. Moreover the long term prosthetic complications and the total costs out‐weighed the differences.^99^ A long‐term retrospective study evaluated the performance of 2‐3 bone‐level implants supporting either three nonsplinted crowns, three splinted crowns, or a three‐unit implant‐supported bridge over two implants. Comparing the three‐unit implant‐supported bridge with one implant less with either the nonsplinted or splinted crowns yielded a reduction in initial costs of 16%. Furthermore, this reduction increased over the duration of the study because the complications were substantially higher in the nonsplinted crowns group.^100^

Despite the increasing levels of evidence in this field of dental research, adjunctive costs‐ related analyses should be encouraged, focusing particularly on the costs related to biologic and technical complications.

## 4 SUMMARY AND CONCLUSIONS

Oral health‐related quality of life has become an important parameter for the assessment of treatment outcomes following implant therapy. However, there is no consensus on the definitions and standardization of this evaluation tool. The discrepancies in the terms, questionnaires, and scales presented in recent decades render a comparison of data challenging. Consequently, further studies with standardized questionnaires are necessary in the future. Nevertheless, the current evidence on function, esthetics, and cost‐effectiveness indicates that:

1. There is evidence that implant‐supported reconstructions have substantially improved the retention and stability of conventional dentures and, hence, enable better chewing and speaking ability of the patient.
2. The connection of implants to prostheses with either locators or balls indicated high oral health‐related quality of life.
3. Patient expectation is not a good predictor of treatment outcome.
4. There is no convincing evidence that oral health‐related quality of life is improved by implant therapy compared with conventional bridge work.
5. In general, there is poor agreement between patients' perceptions and clinicians' objective assessments of esthetic outcomes.
6. There are no significant differences found between the esthetic oral health‐related quality of life ratings for soft tissue‐level implants compared to those for bone‐level implants.
7. Comparison of all‐ceramic and metal‐ceramic restorations showed no significant differences in patients' perceptions in terms of esthetic outcomes.
8. Depending on the choice of outcome measure and financial marginal value, supporting a conventional removable partial denture with implants is cost‐effective when the patient is willing to pay more for achieving a higher level of oral health‐related quality of life.

## 5 CLINICAL CASES

### 5.1 Clinical case 1

The following clinical case illustrates how the replacement of the missing tooth 46 with an implant‐supported single unit crown increased the patient’s quality of life (Figures 1, 2, 3, 4, 5, 6, 7, 8, 9, 10, 11). (Treatment: Dr. Ho‐Yan Duong, Department of Periodontology, University of Bern, Switzerland).

**FIGURE 1:**
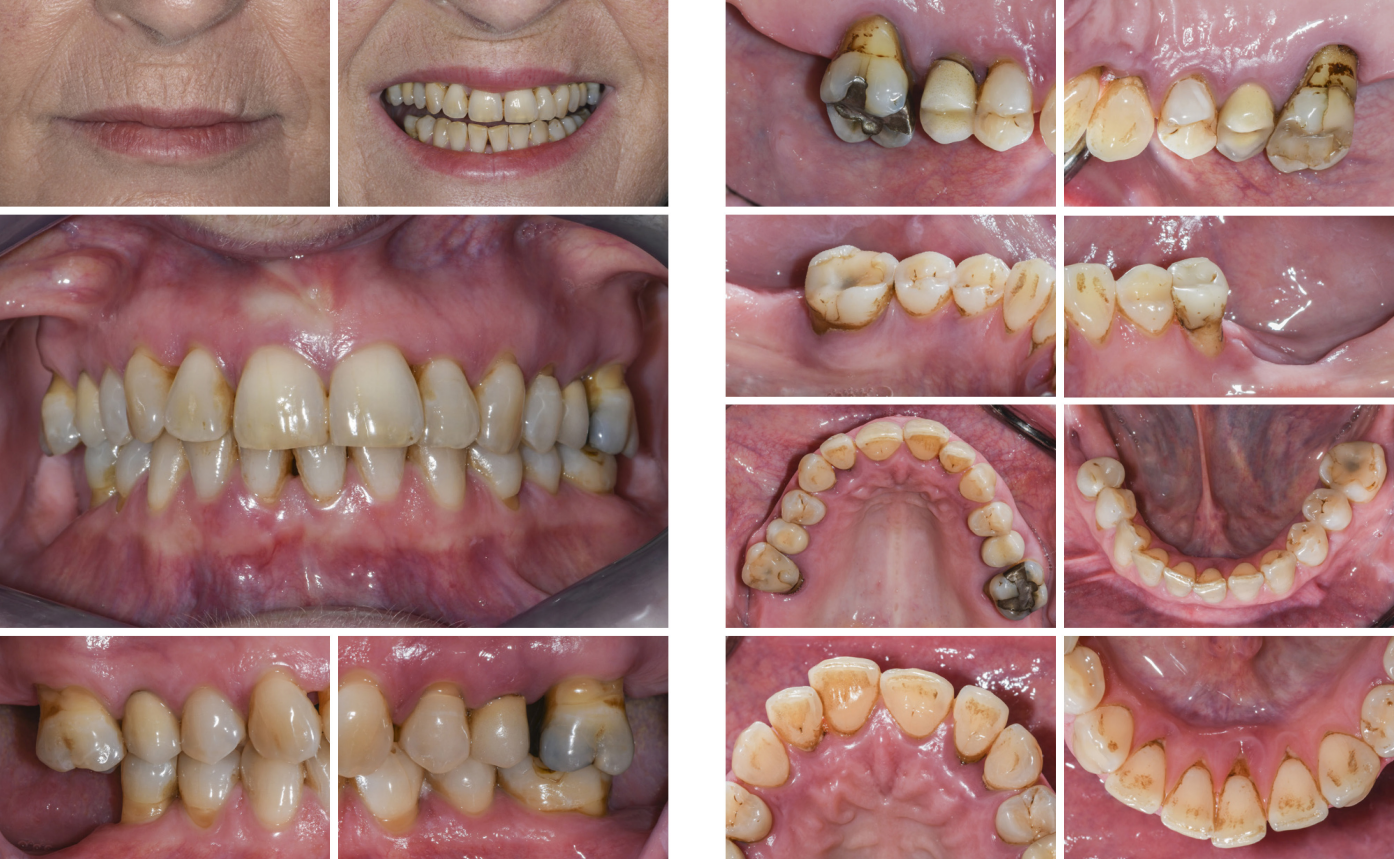
Clinical view at baseline (ie, before steps 1 and 2 of periodontal therapy)

**FIGURE 2:**
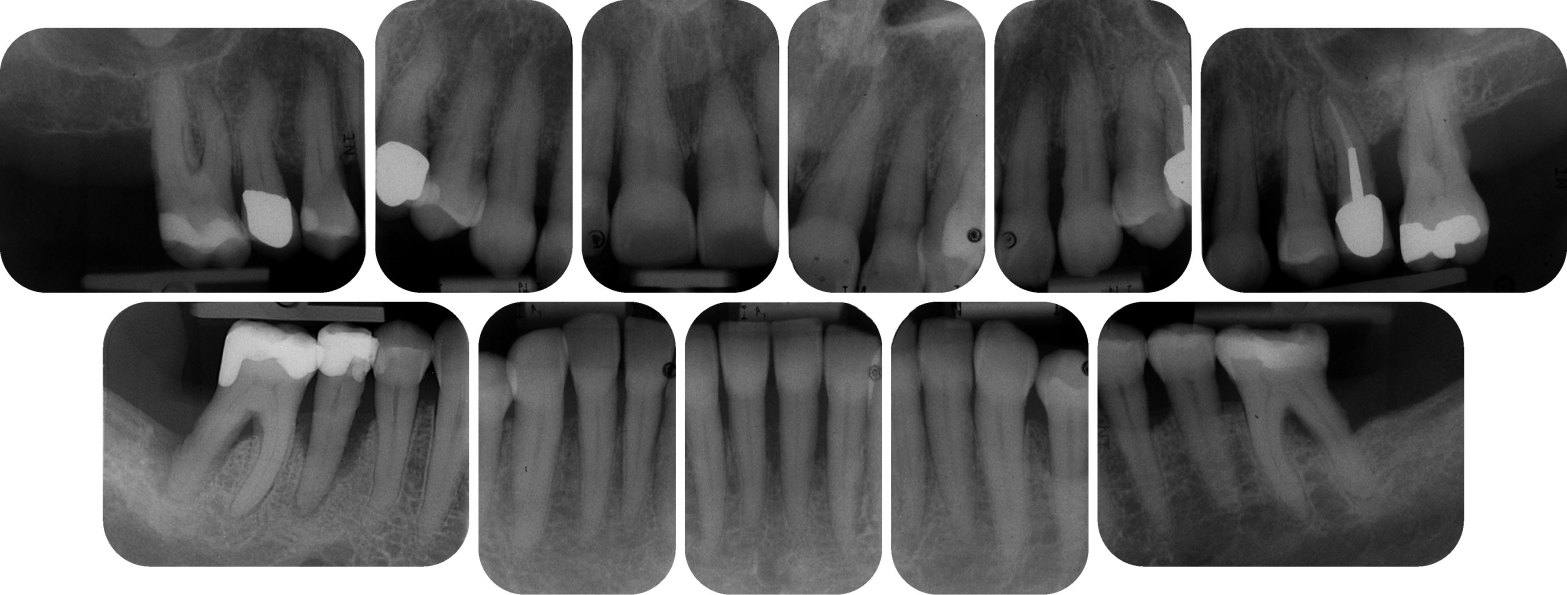
Radiographic view at baseline (ie, before steps 1 and 2 of periodontal therapy) depicting severe horizontal and vertical bone loss

**FIGURE 3:**
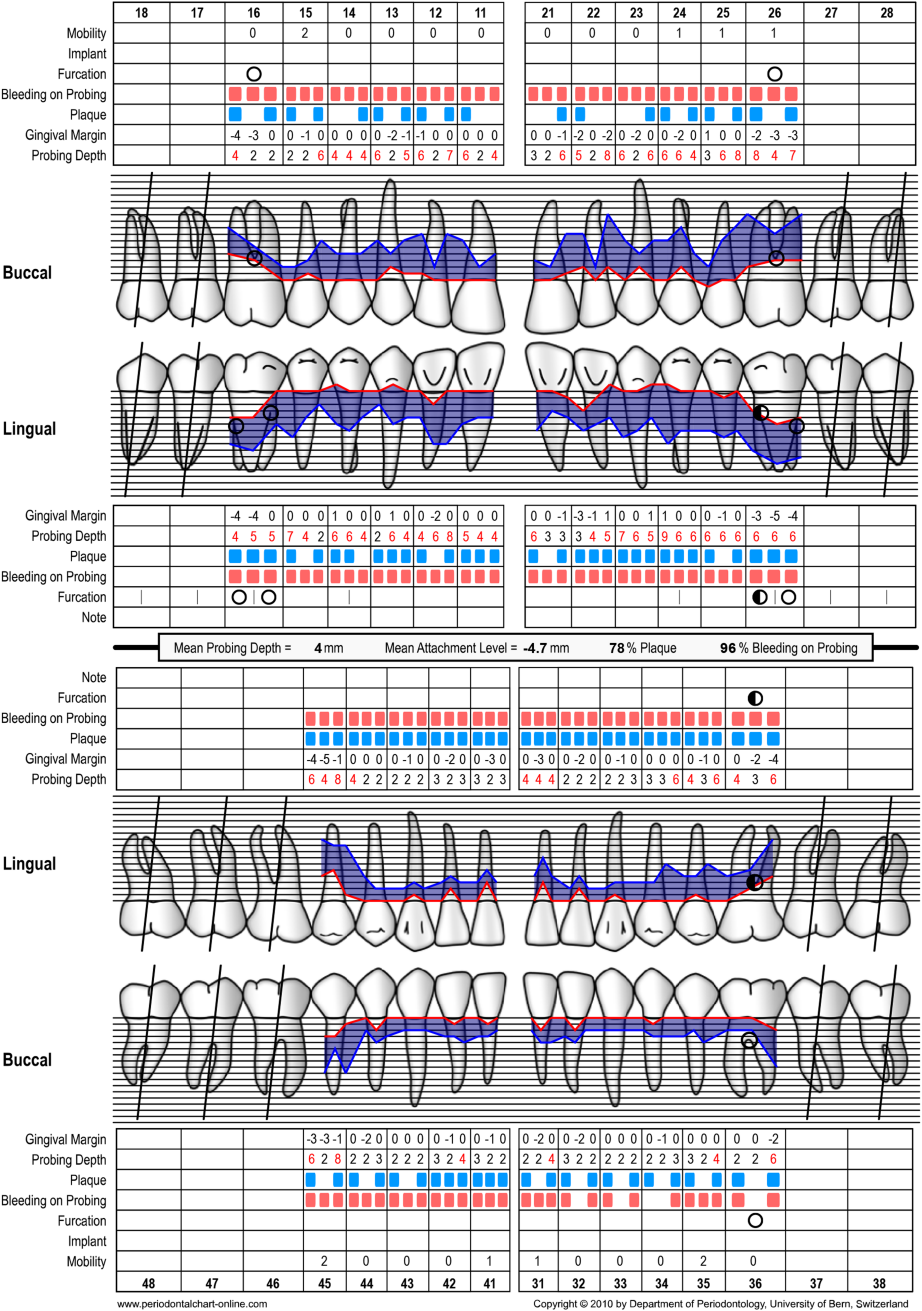
Periodontal chart at baseline (ie, steps 1 and 2 of periodontal therapy) after extraction of tooth 46

**FIGURE 4:**
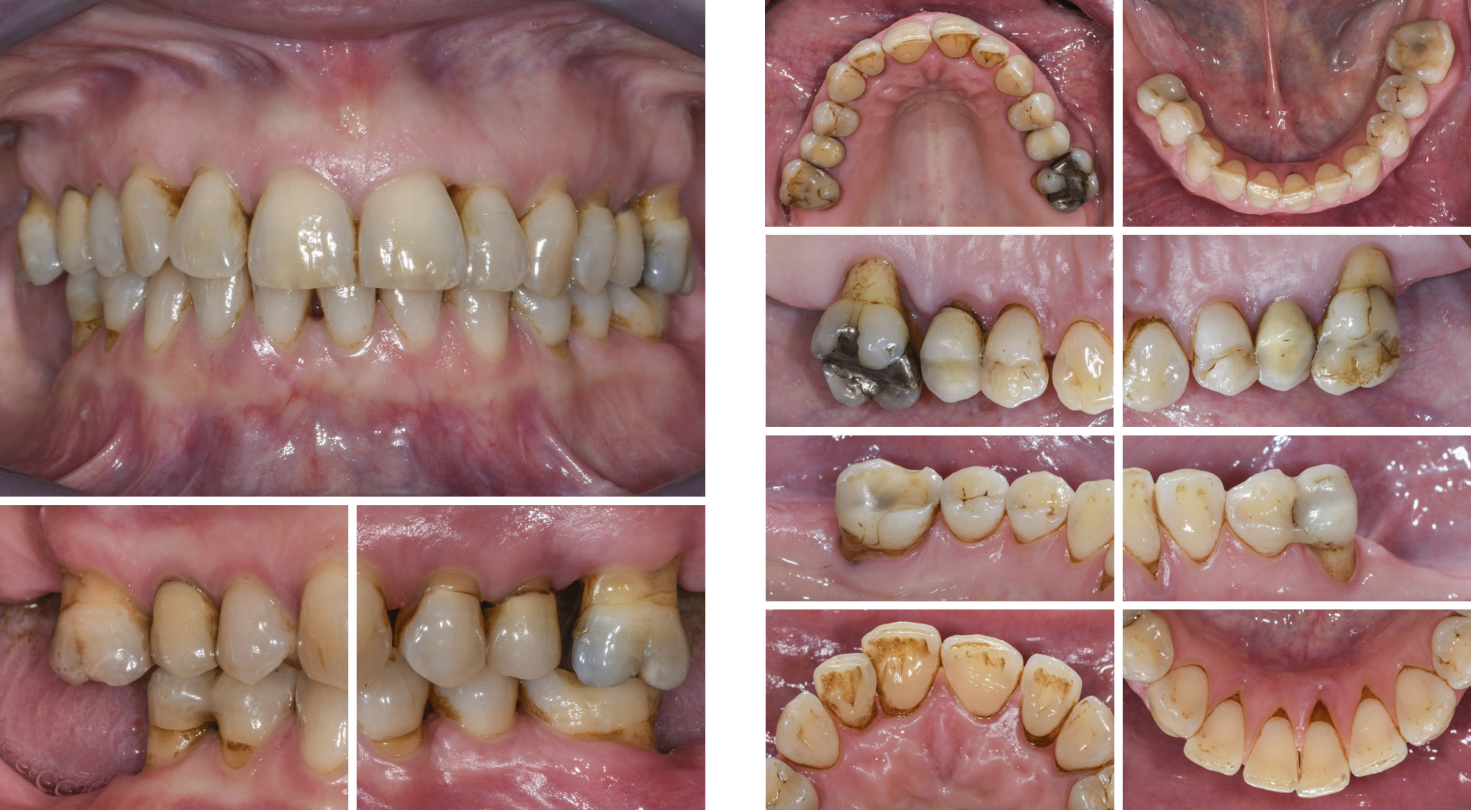
Clinical view at 3 months following nonsurgical periodontal therapy (ie, step 2 of periodontal therapy)

**FIGURE 5:**
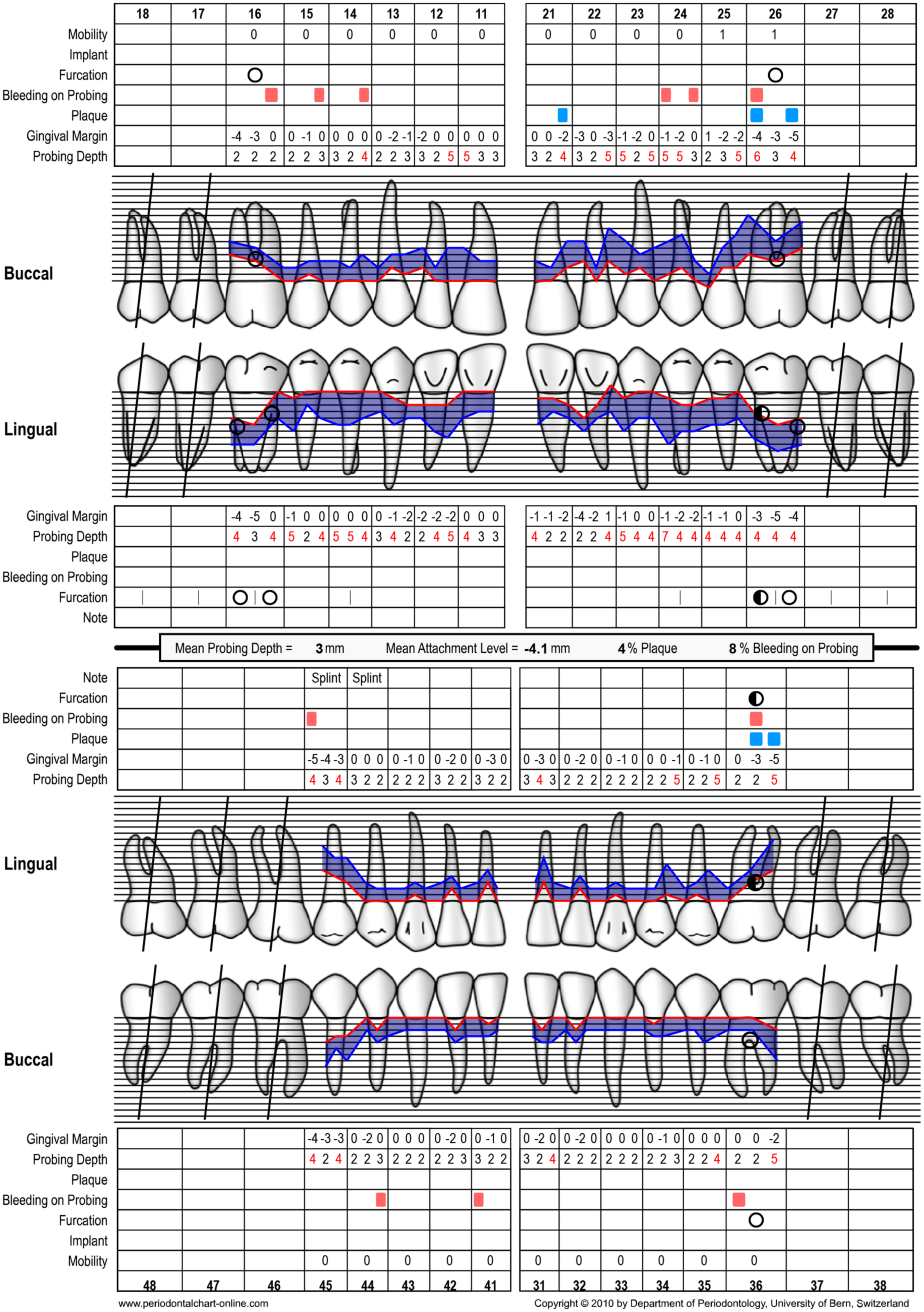
Periodontal chart after nonsurgical periodontal therapy (ie, step 2 of periodontal therapy, after 3 months)

**FIGURE 6:**
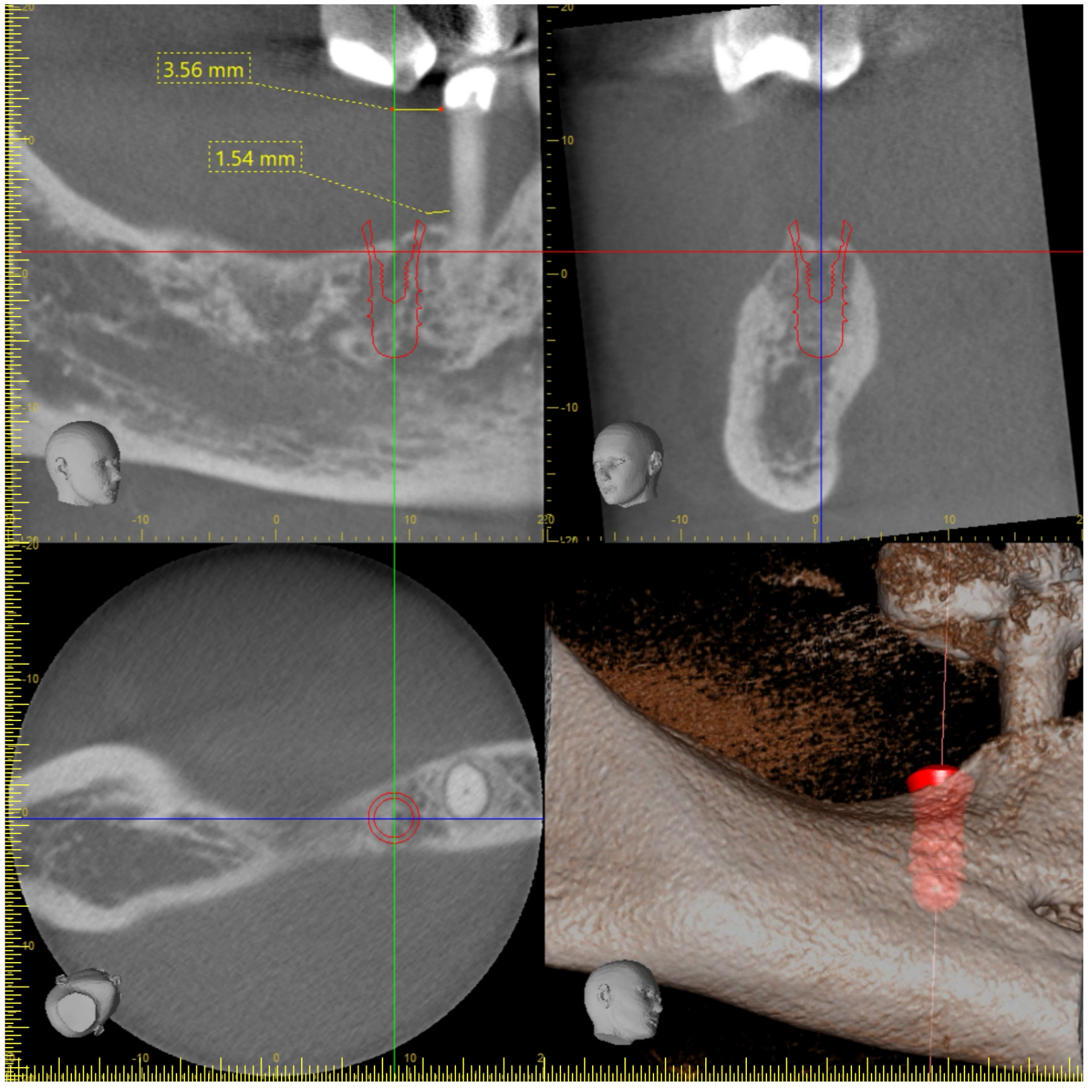
Cone beam computed tomography planning for implant placement in the area of 46

**FIGURE 7:**
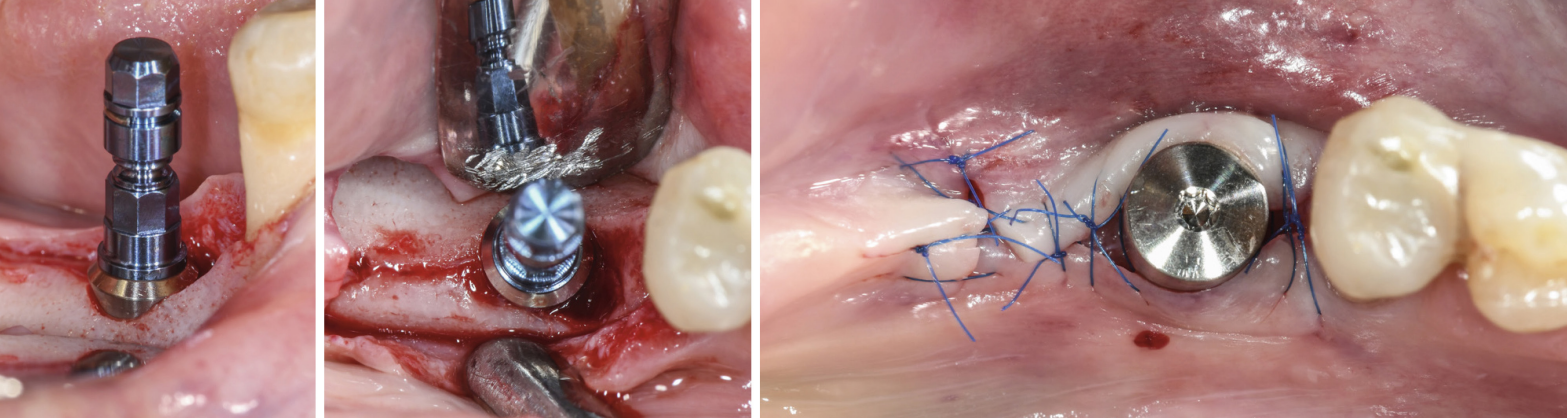
Intraoperative situation depicting implant placement

**FIGURE 8:**
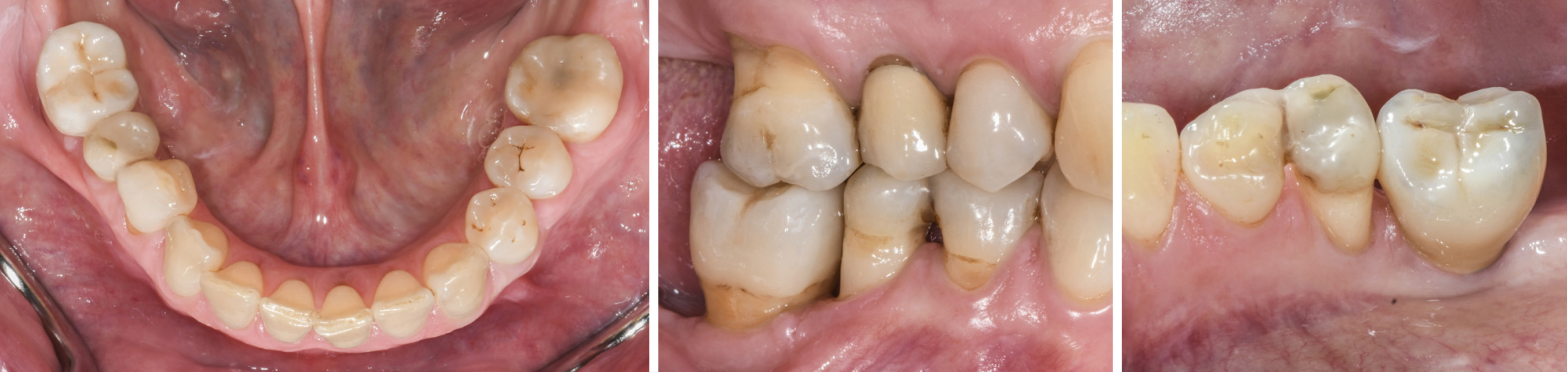
Final situation after delivery of the screw‐retained single unit crown

**FIGURE 9:**
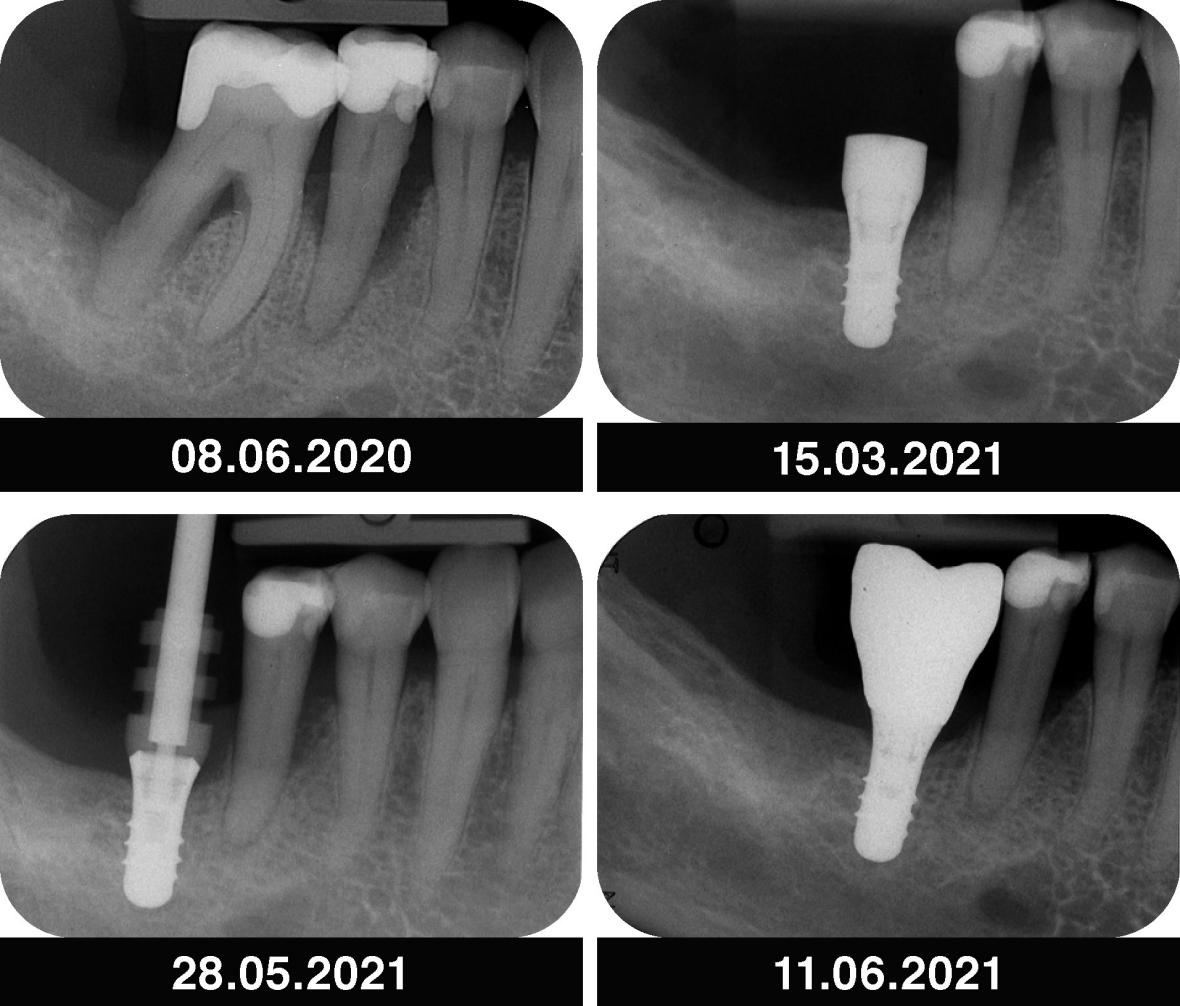
Radiographic images depicting the baseline situation and after implant placement and prosthetic restoration

**FIGURE 10:**
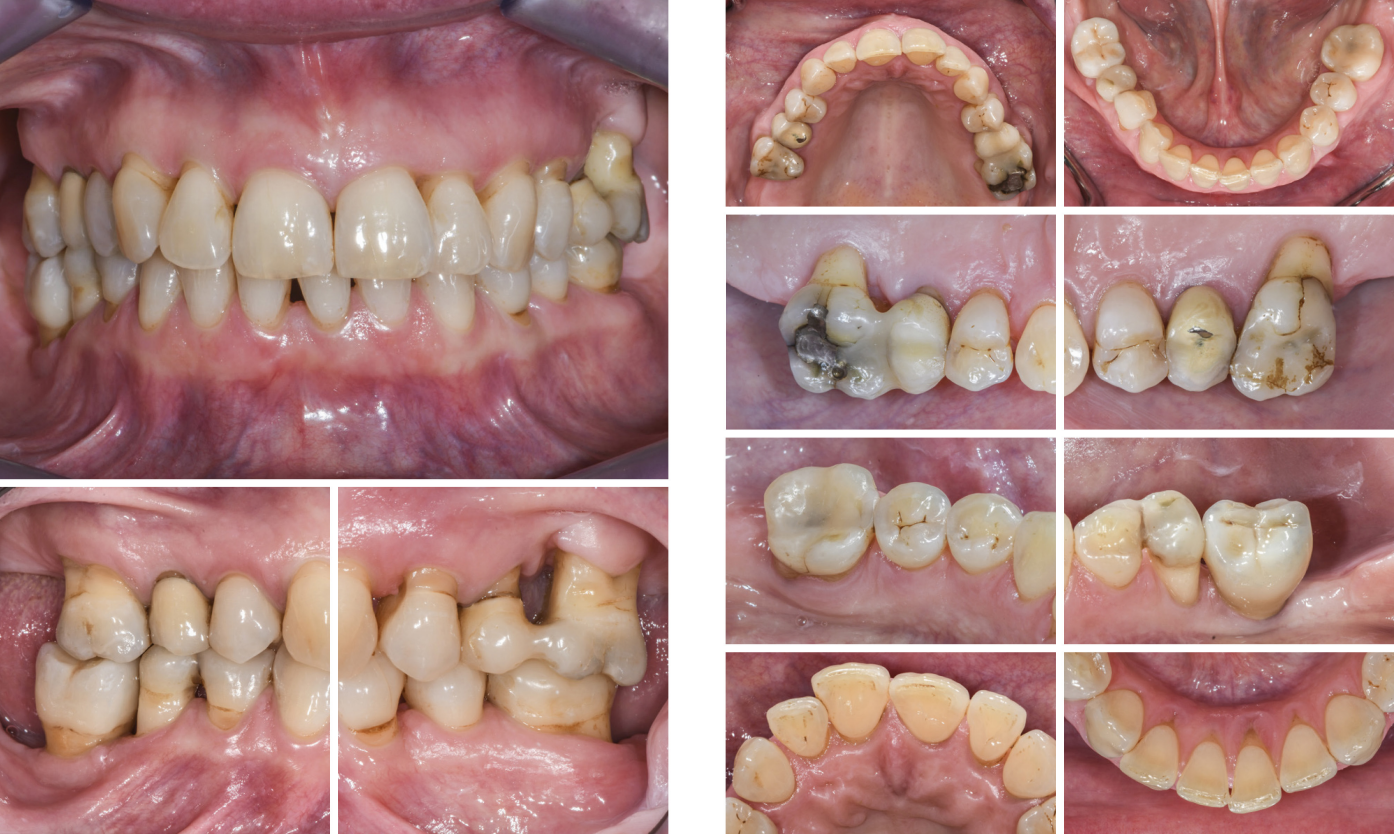
Clinical situation after periodontal therapy, implant placement, and prosthetic restoration

**FIGURE 11:**
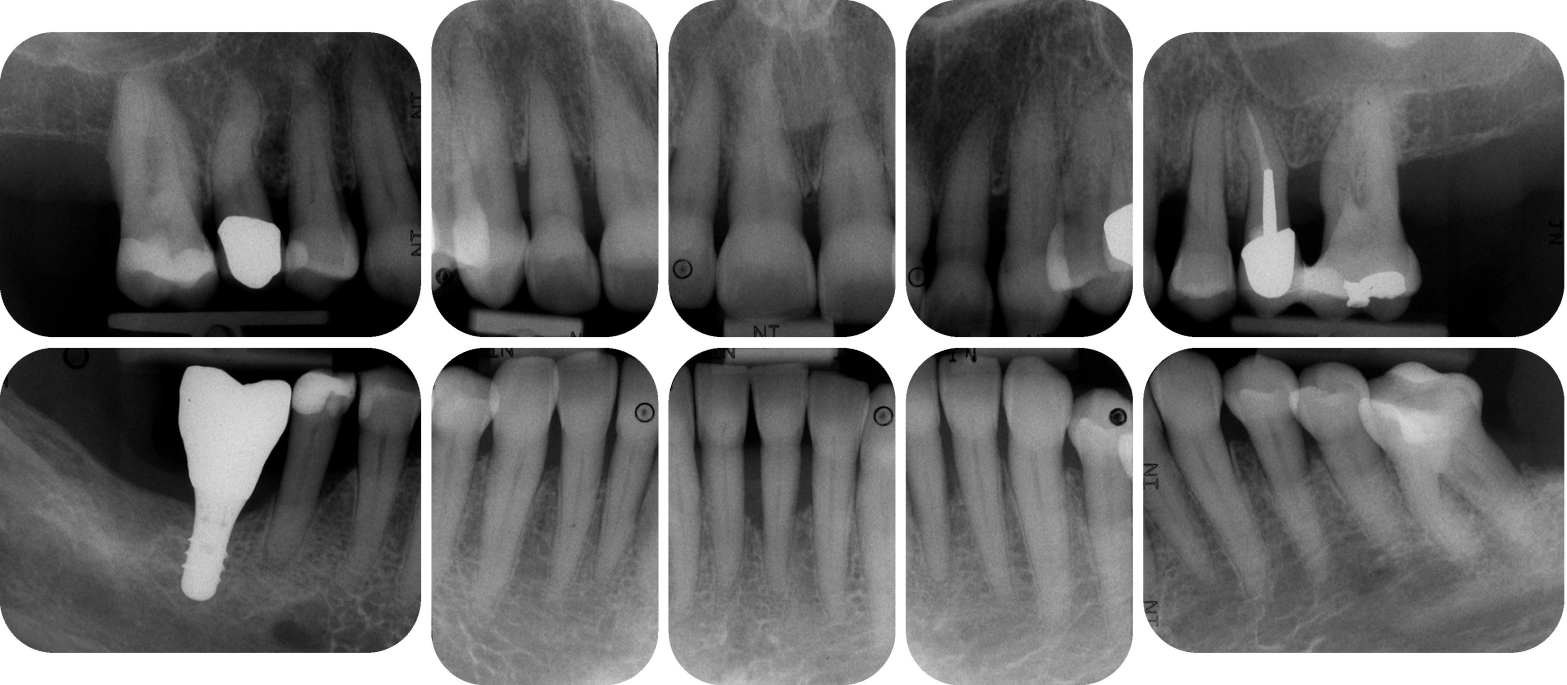
Radiographic view after periodontal therapy, implant placement, and prosthetic restoration indicating healthy periodontal and peri‐implant conditions

A 58‐year‐old female, systemically healthy patient was referred for treatment of advanced periodontal disease. Following the initial clinical and radiographic examination, the following diagnoses were made:

1. generalized periodontitis: stage III, grade B
2. bruxism
3. trauma from occlusion
4. endo‐periodontal lesion with root damage

**FIGURE 12:**
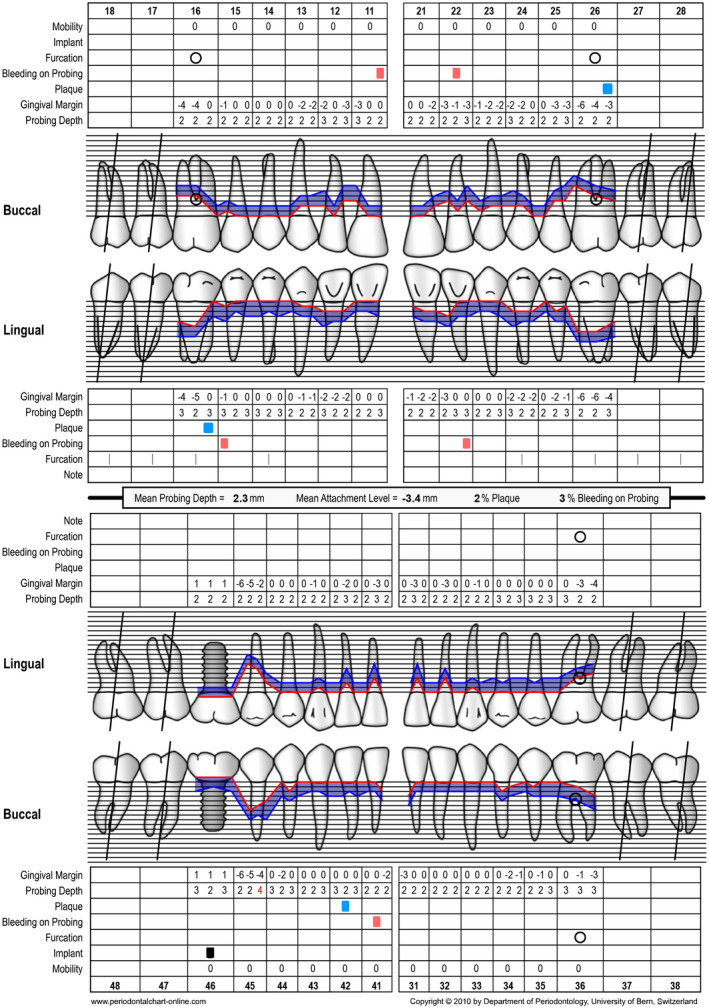
Periodontal chart after periodontal therapy, implant placement, and prosthetic restoration indicating healthy periodontal and peri‐implant conditions

Tooth 46 had to be extracted before steps 1 and 2 of periodontal therapy^101^ because of a large endo‐perio lesion. A re‐evaluation was performed at 3 and at 6 months after step 2 of periodontal therapy (ie, full‐mouth subgingival scaling and root planing). Before implant placement, no probing depths exceeding 5 mm were recorded. Thereafter, the missing tooth 46 was replaced with an implant‐supported restoration by accommodating a soft tissue‐level implant (Straumann: TL, SP, ø 4.1 mm, SLActive, 8 mm, Roxolid) followed by transmucosal healing. The patient did not receive any provisional prosthesis during the healing period of 3 months. After 3 months, the implant was loaded with a full zirconia screw‐retained crown. Tooth 45 did not show any pathologic symptoms during the entire treatment. Periodontal chart of final examination is depicted in figure 12.

After prosthetic restoration, the patient was asked to compete the oral health impact profile‐14 questionnaire (Table 4).

**TABLE 4:** Oral health impact profile‐14 questionnaire filled out 2 mo after restoration (case 1)

	Question	Very often	Fairly often	Occasionally	Hardly ever	Never	Dimension	Weight
1	Have you had trouble pronouncing any words because of problems with your teeth, mouth, or dentures?					x	Functional limitation	0.51
2	Have you felt that your sense of taste has worsened because of problems with your teeth, mouth, or dentures?					x	Functional limitation	0.49
3	Have you had painful aching in your mouth?					x	Physical pain	0.34
4	Have you found it uncomfortable to eat any foods because of problems with your teeth, mouth, or dentures?					x	Physical pain	0.66
5	Have you been self‐conscious because of your teeth, mouth, or dentures?					x	Psychological discomfort	0.45
6	Have you felt tense because of problems with your teeth, mouth, or dentures?					x	Psychological discomfort	0.55
7	Has your diet been unsatisfactory because of problems with your teeth, mouth, or dentures?					x	Physical disability	0.52
8	Have you had to interrupt meals because of problems with your teeth, mouth, or dentures?					x	Physical disability	0.48
9	Have you found it difficult to relax because of problems with your teeth, mouth, or dentures?					x	Psychological disability	0.60
10	Have you been a bit embarrassed because of problems with your teeth, mouth, or dentures?					x	Psychological disability	0.40
11	Have you been a bit irritable with other people because of problems with your teeth, mouth, or dentures?					x	Social disability	0.62
12	Have you had difficulty doing your usual jobs because of problems with your teeth, mouth, or dentures?					x	Social disability	0.38
13	Have you felt that life in general was less satisfying because of problems with your teeth, mouth, or dentures?					x	Handicap	0.59
14	Have you been totally unable to function because of problems with your teeth, mouth, or dentures?					x	Handicap	0.41

According to Slade & Spencer,^22^ the responses are coded 0 = never, 1 = hardly ever, 2 = occasionally, 3 = fairly often, and 4 = very often. The coded responses can be subdivided into seven dimensions (functional limitation, physical pain, psychological discomfort, physical disability, psychological disability, social disability, and handicap). Within each dimension, coded responses were multiplied with specific weights. The result of the response was 0, showing the highest possible score for quality of life according to this questionnaire and this treatment modality.

### 5.2 Clinical case 2

The following clinical case illustrates how the replacement of the missing tooth 46 with an implant‐supported single unit crown increased the patient's quality of life (Figures 13, 14, 15, 16, 17, 18, 19, 20). (Treatment: Dr. Ho‐Yan Duong, Department of Periodontology, University of Bern, Switzerland).

**FIGURE 13:**
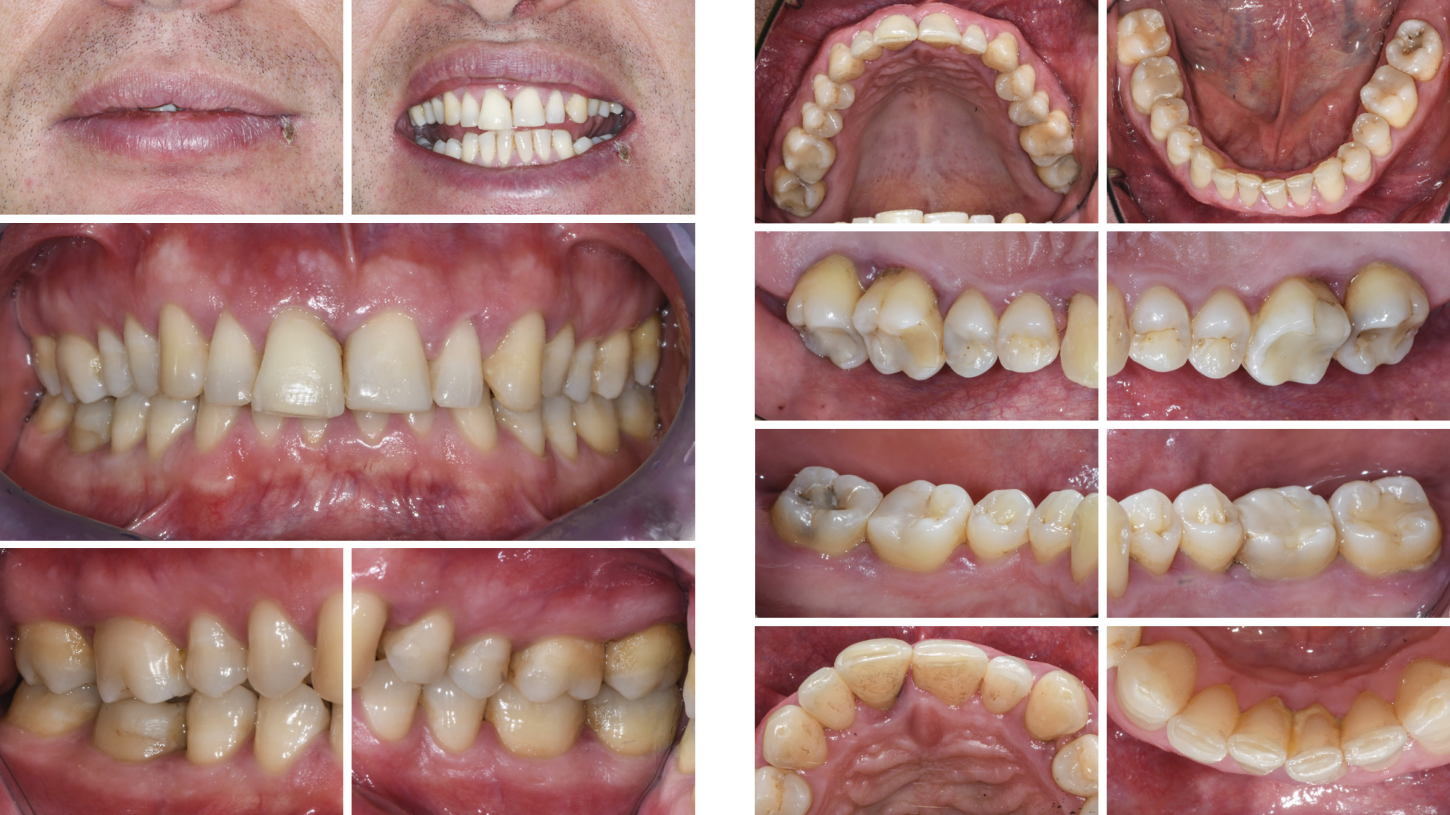
Clinical view at baseline (ie, before steps 1 and 2 of periodontal therapy)

**FIGURE 14:**
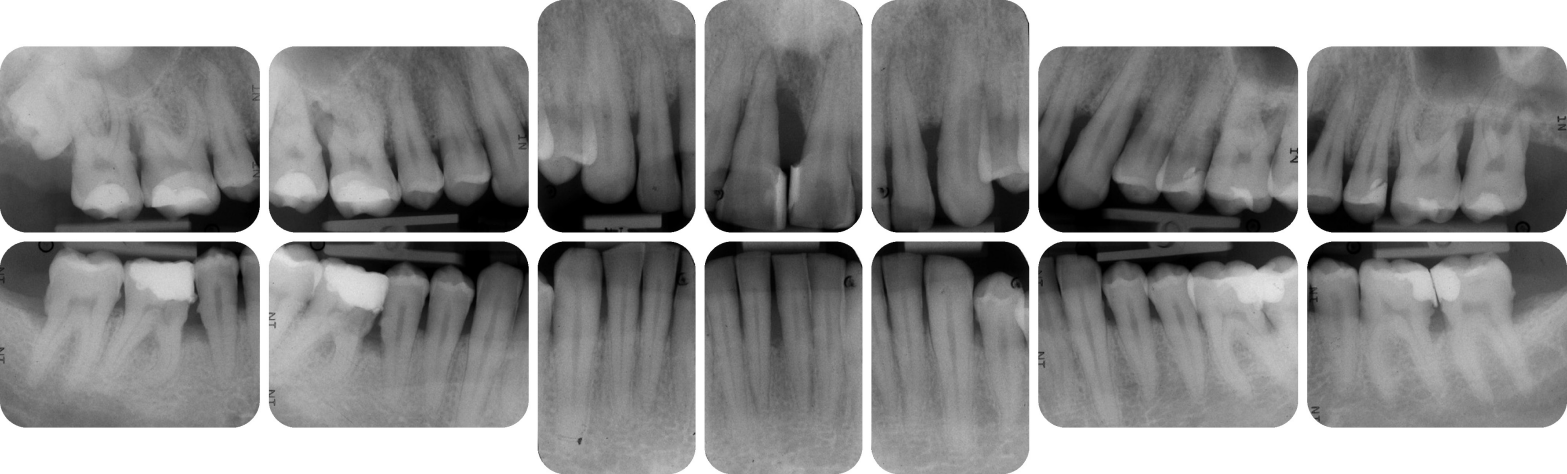
Radiographic images at baseline (ie, before steps 1 and 2 of periodontal therapy)

**FIGURE 15:**
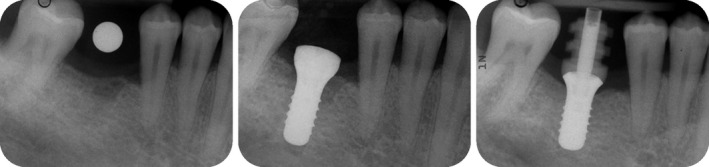
Radiographic planning for implant placement in the area of 46

**FIGURE 16:**
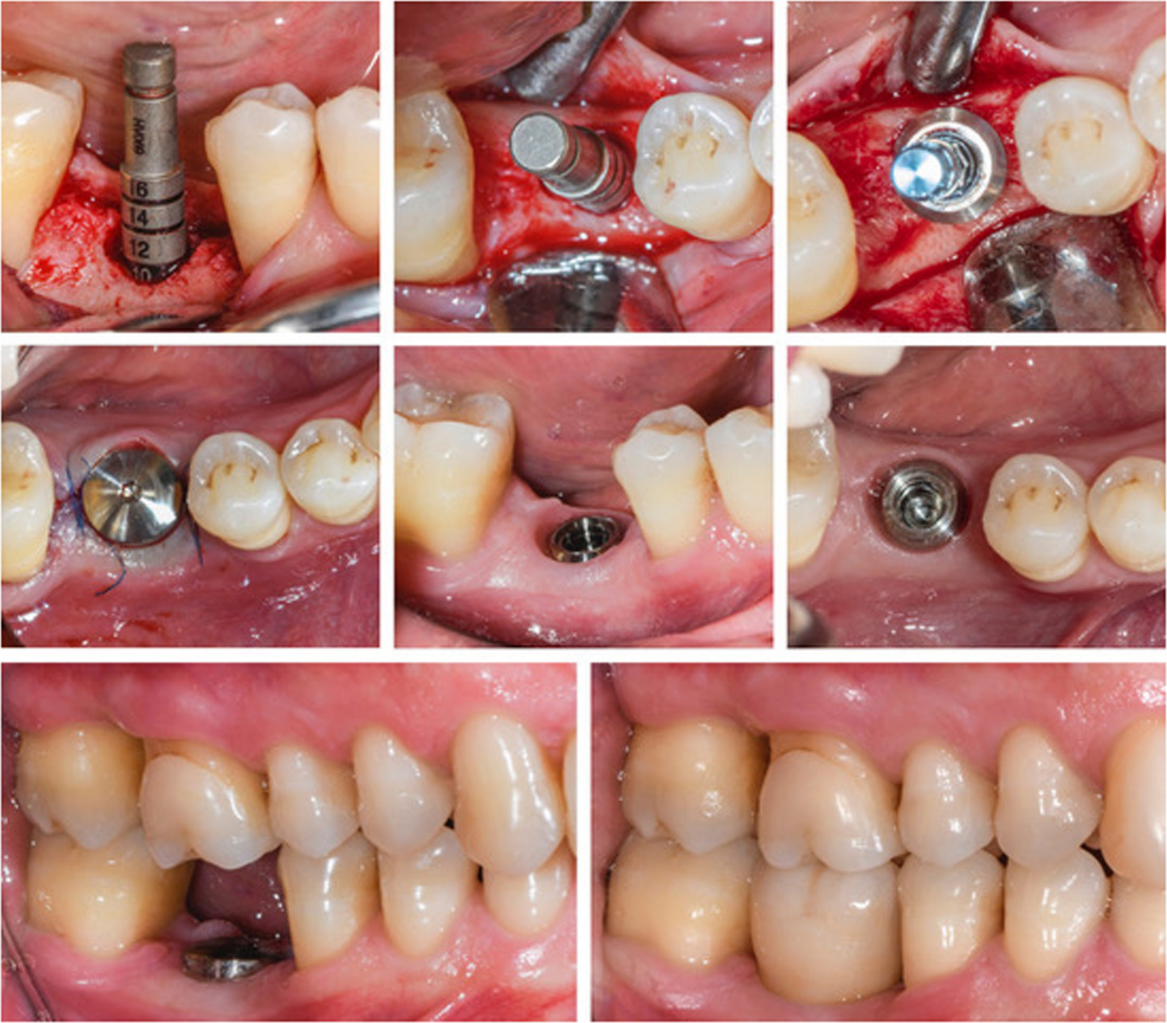
Intraoperative situation depicting implant placement, healing phase and delivery of the prosthetic restoration

**FIGURE 17:**
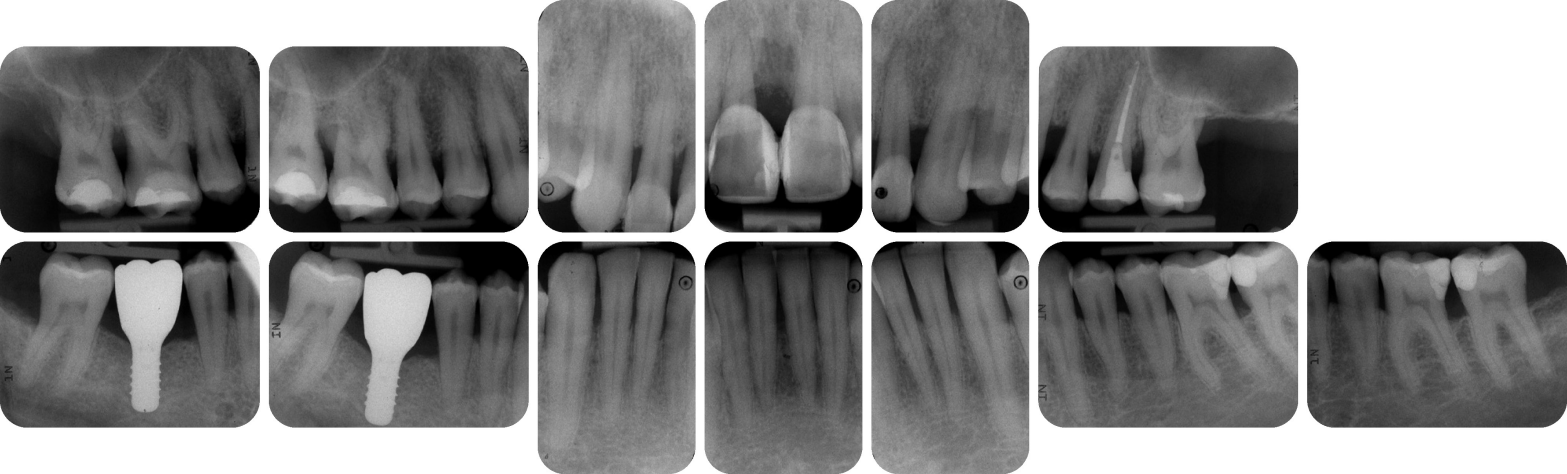
Radiographic view after periodontal therapy, implant placement, and prosthetic restoration indicating healthy periodontal and peri‐implant conditions

**FIGURE 18:**
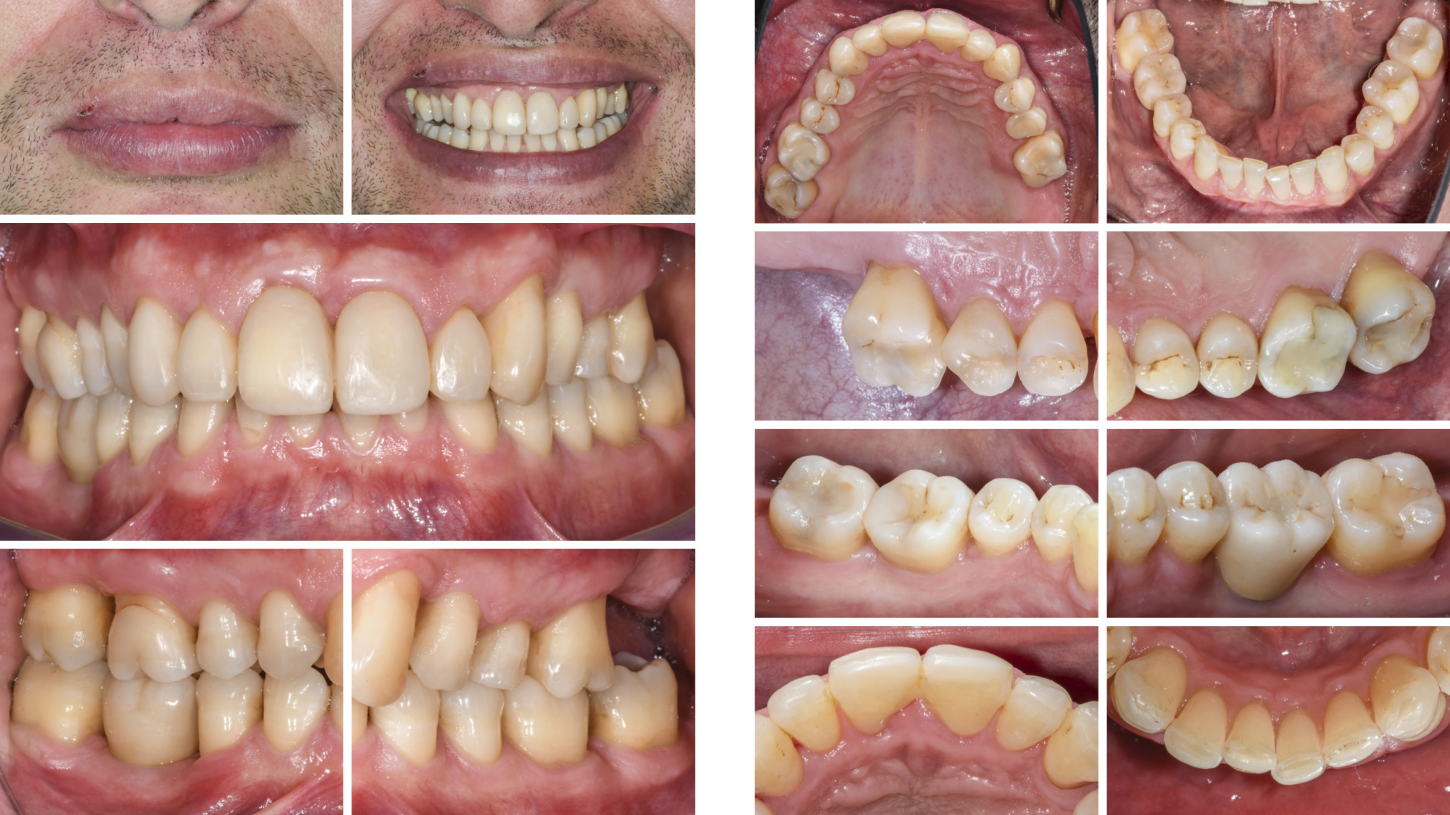
Clinical situation after periodontal therapy, implant placement, and prosthetic restoration

**FIGURE 19:**
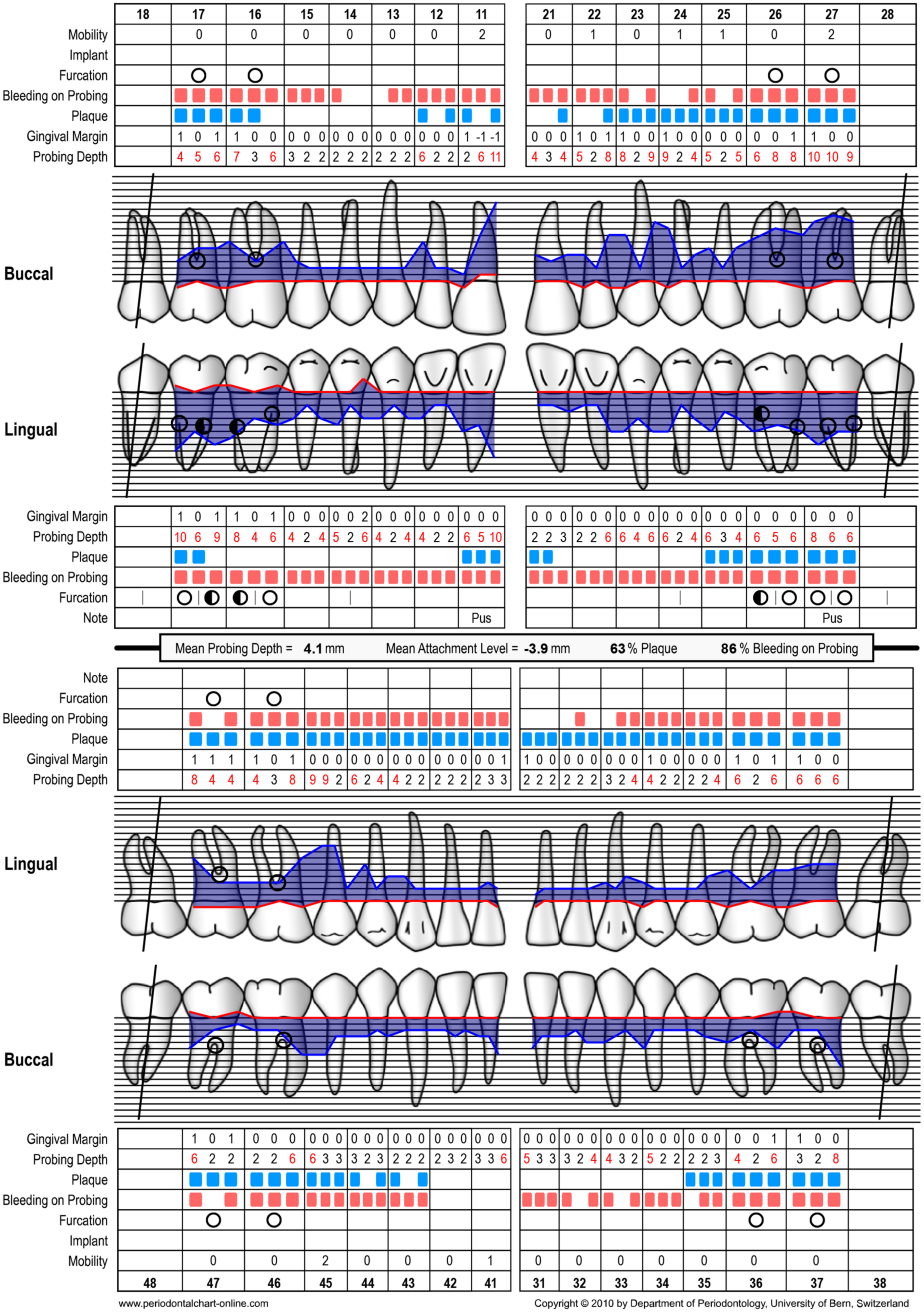
Periodontal chart at baseline (ie, before steps 1 and 2 of periodontal therapy) before extraction of tooth 46

**FIGURE 20:**
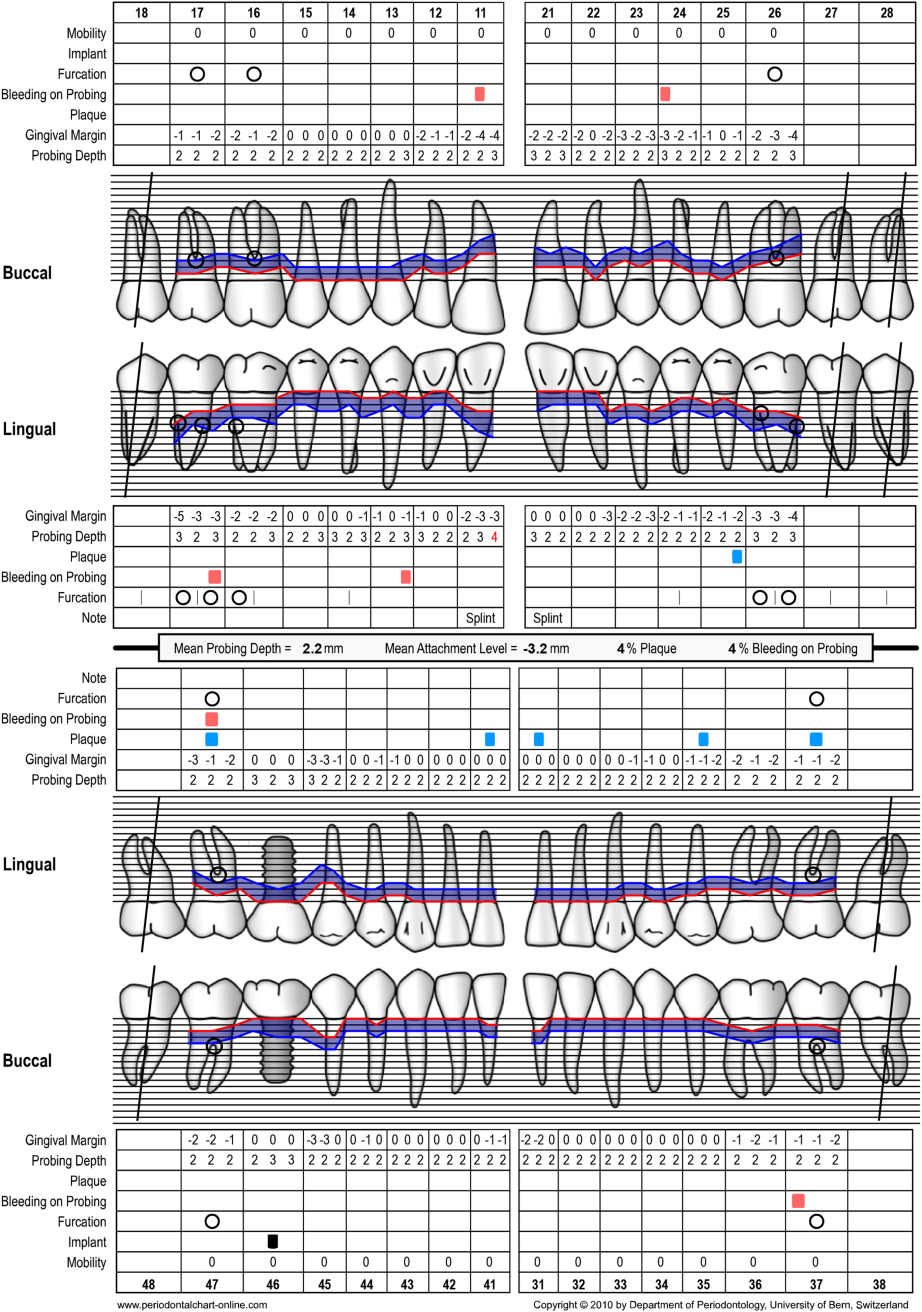
Periodontal chart after periodontal therapy, implant placement, and prosthetic restoration indicating healthy periodontal and peri‐implant conditions

A 41‐year‐old male, systemically healthy patient was referred for treatment of advanced periodontal disease. Following initial clinical and radiographic examination, the following diagnoses were made:

1. generalized periodontitis: stage III, grade B
2. endo‐periodontal lesion without root damage
3. caries

Tooth 46 had to be extracted before steps 1 and 2 of periodontal therapy^101^ because of the extension of caries into the furcation. A re‐evaluation was performed at 3 months and at 6 months after step 2 of periodontal therapy (ie, full‐mouth subgingival scaling and root planing). Before implant placement, no probing depths exeeding 5 mm were recorded. Thereafter, the missing tooth 46 was replaced with an implant‐supported restoration by accommodating a soft tissue‐level implant (Straumann: TL, SP, ø 4.8 mm WN, SLActive, 8 mm, Roxolid) followed by transmucosal healing. The patient did not receive any provisional prosthesis during the healing period of 3 months. After 3 months, the implant was loaded with a full zirconia screw‐retained crown. After prosthetic restoration, the patient was asked to complete the oral health impact profile‐14 questionnaire (Table 5).

According to Slade & Spencer,^22^ the result of the response was 0, showing the highest possible score for quality of life according to this questionnaire and this treatment modality (Table 5).

**TABLE 5:** Oral health impact profile‐14 questionnaire filled out 2 mo after restoration (case 2)

	Question	Very often	Fairly often	Occasionally	Hardly ever	Never	Dimension	Weight
1	Have you had trouble pronouncing any words because of problems with your teeth, mouth, or dentures?					x	Functional limitation	0.51
2	Have you felt that your sense of taste has worsened because of problems with your teeth, mouth, or dentures?					x	Functional limitation	0.49
3	Have you had painful aching in your mouth?					x	Physical pain	0.34
4	Have you found it uncomfortable to eat any foods because of problems with your teeth, mouth, or dentures?					x	Physical pain	0.66
5	Have you been self‐conscious because of your teeth, mouth, or dentures?					x	Psychological discomfort	0.45
6	Have you felt tense because of problems with your teeth, mouth, or dentures?					x	Psychological discomfort	0.55
7	Has your diet been unsatisfactory because of problems with your teeth, mouth, or dentures?					x	Physical disability	0.52
8	Have you had to interrupt meals because of problems with your teeth, mouth, or dentures?					x	Physical disability	0.48
9	Have you found it difficult to relax because of problems with your teeth, mouth, or dentures?					x	Psychological disability	0.60
10	Have you been a bit embarrassed because of problems with your teeth, mouth, or dentures?					x	Psychological disability	0.40
11	Have you been a bit irritable with other people because of problems with your teeth, mouth, or dentures?					x	Social disability	0.62
12	Have you had difficulty doing your usual jobs because of problems with your teeth, mouth, or dentures?					x	Social disability	0.38
13	Have you felt that life in general was less satisfying because of problems with your teeth, mouth, or dentures?					x	Handicap	0.59
14	Have you been totally unable to function because of problems with your teeth, mouth, or dentures?					x	Handicap	0.41

## References

[1] Karoussis I , Salvi G , Heitz‐Mayfield L , Brägger U , Hämmerle C , Lang NP . Long‐term implant prognosis in patients with and without a history of chronic periodontitis: a 10‐year prospective cohort study of the ITI Dental Implant System. Clin Oral Implants Res. 2003;14:329‐339. 12755783 10.1034/j.1600-0501.000.00934.x

[2] Brånemark PI , Adell R , Breine U , Hansson BO , Lindström J , Ohlsson A . Intra‐osseous anchorage of dental prostheses. I. Experimental studies. Scand J Plast Reconstr Surg. 1969;3:81‐100. 4924041 10.3109/02844316909036699

[3] Adell R , Lekholm U , Rockler B , Branemark PI . A 15‐year study of osseointegrated implants in the treatment of the edentulous jaw. Int J Oral Surg. 1981;10:387‐416. 6809663 10.1016/s0300-9785(81)80077-4

[4] Chen ST , Wilson TG Jr , Hämmerle CH . Immediate or early placement of implants following tooth extraction: review of biologic basis, clinical procedures, and outcomes. Int J Oral Maxillofac Implants. 2004;19(Suppl):12‐25. 15635942

[5] Gallucci GO , Hamilton A , Zhou W , Buser D , Chen S . Implant placement and loading protocols in partially edentulous patients: a systematic review. Clin Oral Implants Res. 2018;29(Suppl 16):106‐134. 30328194 10.1111/clr.13276

[6] Bornstein MM , Halbritter S , Harnisch H , Weber HP , Buser D . A retrospective analysis of patients referred for implant placement to a specialty clinic: indications, surgical procedures, and early failures. Int J Oral Maxillofac Implants. 2008;23:1109‐1116. 19216281

[7] Brügger OE , Bornstein MM , Kuchler U , Janner SFM , Chappuis V , Buser D . Implant therapy in a surgical specialty clinic: an analysis of patients, indications, surgical procedures, risk factors, and early failures. Int J Oral Maxillofac Implants. 2015;30:151‐160. 25506641 10.11607/jomi.3769

[8] Belser UC , Buser D , Hess D , Schmid B , Bernard JP , Lang NP . Aesthetic implant restorations in partially edentulous patients–a critical appraisal. Periodontol 2000. 1998;17:132‐150. 10337321 10.1111/j.1600-0757.1998.tb00131.x

[9] Chen ST , Buser D . Esthetic outcomes following immediate and early implant placement in the anterior maxilla–a systematic review. Int J Oral Maxillofac Implants. 2014;29(Suppl):186‐215. 24660198 10.11607/jomi.2014suppl.g3.3

[10] Hämmerle CH , Chen ST , Wilson TG Jr . Consensus statements and recommended clinical procedures regarding the placement of implants in extraction sockets. Int J Oral Maxillofac Implants. 2004;19(Suppl):26‐28. 15635943

[11] Morton D , Chen ST , Martin WC , Levine RA , Buser D . Consensus statements and recommended clinical procedures regarding optimizing esthetic outcomes in implant dentistry. Int J Oral Maxillofac Implants. 2014;29(Suppl):216‐220. 24660199 10.11607/jomi.2013.g3

[12] Calvert M , Kyte D , Mercieca‐Bebber R , et al. Guidelines for inclusion of patient‐reported outcomes in clinical trial protocols: the SPIRIT‐PRO extension. JAMA. 2018;319:483‐494. 29411037 10.1001/jama.2017.21903

[13] Wyrwich KW , Norquist JM , Lenderking WR , Acaster S . & Industry Advisory Committee of International Society for Quality of Life, R. Methods for interpreting change over time in patient‐reported outcome measures. Qual Life Res. 2013;22:475‐483. 22528240 10.1007/s11136-012-0175-x

[14] De Bruyn H , Raes S , Matthys C , Cosyn J . The current use of patient‐centered/reported outcomes in implant dentistry: a systematic review. Clin Oral Implants Res. 2013;26(Suppl 11):45‐56. 10.1111/clr.12634 26385620

[15] Lang NP , Zitzmann NU & Working Group 3 of the VIII European Workshop on Periodontology . Clinical research in implant dentistry: evaluation of implant‐supported restorations, aesthetic and patient‐reported outcomes. J Clin Periodontol 2012;39(Suppl 12):133‐138. 22533953 10.1111/j.1600-051X.2011.01842.x

[16] Slade GD , Strauss RP , Kressin NR , Locker D , Reisine ST. Conference summary: assessing oral health outcomes – measuring health status and quality of life. Community Dent Health. 1998; 15(1): 3‐7. 9791607

[17] Locker D , Allen F . What do measures of 'oral health‐related quality of life' measure? Community Dent Oral Epidemiol. 2007;35:401‐411. 18039281 10.1111/j.1600-0528.2007.00418.x

[18] Grant S , Aitchison T , Henderson E , et al. A comparison of the reproducibility and the sensitivity to change of visual analogue scales, Borg scales, and Likert scales in normal subjects during submaximal exercise. Chest. 1999;116:1208‐1217. 10559077 10.1378/chest.116.5.1208

[19] Reville SI , Robinson JO , Rosen M , Hogg MIJ . Reliability of linear analogue scales for evaluation of pain. Anaesthesia. 1976;31:1191‐1198. 1015603 10.1111/j.1365-2044.1976.tb11971.x

[20] Likert R . A technique for the measurement of attitudes. Archiv Psychol. 1932;140:1‐55.

[21] Slade GD . Derivation and validation of a short‐form oral health impact profile. Community Dent Oral Epidemiol. 1997;25:284‐290. 9332805 10.1111/j.1600-0528.1997.tb00941.x

[22] Slade GD , Spencer AJ . Development and evaluation of the Oral Health Impact Profile. Community Dent Health. 1994;11:3‐11. 8193981

[23] Bennadi D , Reddy CV . Oral health related quality of life. J Int Soc Prev Community Dent. 2013;3:1‐6. 24478972 10.4103/2231-0762.115700 PMC3894098

[24] Cushing AM , Sheiham A , Maizels J . Developing socio‐dental indicators–the social impact of dental disease. Community Dent Health. 1986;3:3‐17. 3516317

[25] Atchison KA , Dolan TA . Development of the Geriatric Oral Health Assessment Index. J Dent Educ. 1990;54:680‐687. 2229624

[26] Strauss RP , Hunt RJ . Understanding the value of teeth to older adults: influences on the quality of life. J Am Dent Assoc. 1993;124:105‐110. 10.14219/jada.archive.1993.0019 8445136

[27] Locker D , Miller Y . Evaluation of subjective oral health status indicators. J Public Health Dent. 1994;54:167‐176. 7932353 10.1111/j.1752-7325.1994.tb01209.x

[28] Leao A , Sheiham A . The development of a socio‐dental measure of dental impacts on daily living. Community Dent Health. 1996;13:22‐26. 8634892

[29] Adulyanon S , Sheiham A . Measuring oral health and quality of life. In: Slade GD , ed. Oral impacts on daily performances. University of North Carolina, Dental Ecology; 1997:152‐160.

[30] Kressin N . Measuring oral health and quality of life. In: Slade GD , ed. The Oral Health Related Quality of Life Measure (OHQOL). University of North Carolina, Dental Ecology; 1997:114‐119.

[31] Cornell J , Saunders M , Paunovich ED , Frisch MB . Measuring oral health and quality of life. In: Slade GD , ed. Oral Health Quality of Life Inventory (OH‐QoL). University of North Carolina, Dental Ecology; 1997:136‐149.

[32] Dolan T , Gooch B . Dental health questions from the Rand Health Insurance Study. In: Slade GD , ed. Measuring Oral Health and Quality of Life. University of North Carolina, Dental Ecology; 1997:66‐70.

[33] Cunningham SJ , Garratt AM , Hunt NP . Development of a condition‐specific quality of life measure for patients with dentofacial deformity: I. reliability of the instrument. Community Dent Oral Epidemiol. 2000;28:195–201.35. 10830646 10.1034/j.1600-0528.2000.280305.x

[34] Jokovic A , Locker D , Stephens M , Kenny D , Tompson B , Guyatt G . Validity and reliability of a questionnaire for measuring child oral‐health‐related quality of life. J Dent Res. 2002;81:459‐463. 12161456 10.1177/154405910208100705

[35] McGrath C , Bedi R . Measuring the impact of oral health on quality of life in Britain using OHQoL‐UK(W). J Public Health Dent. 2003;63:73‐77. 12816136 10.1111/j.1752-7325.2003.tb03478.x

[36] Gherunpong S , Tsakos G , Sheiham A . Developing and evaluating an oral health‐related quality of life index for children; the CHILD‐OIDP. Community Dent Health. 2004;21:161‐169. 15228206

[37] Klages U , Claus N , Wehrbein H , Zentner A . Development of a questionnaire for assessment of the psychosocial impact of dental aesthetics in young adults. Eur J Orthod. 2006;28:103‐111. 16257989 10.1093/ejo/cji083

[38] Locker D , Berka E , Jokovic A , Tompson B . Does self‐weighting of items enhance the performance of an oral health‐related quality of life questionnaire? Community Dent Oral Epidemiol. 2007;35:35‐43. 17244136 10.1111/j.1600-0528.2007.00317.x

[39] Preciado A , Del Rio J , Lynch CD , Castillo‐Oyagüe R . A new, short, specific questionnaire (QoLIP‐10) for evaluating the oral health‐related quality of life of implant‐retained overdenture and hybrid prosthesis wearers. J Dent. 2013;41:753‐763. 23831418 10.1016/j.jdent.2013.06.014

[40] Farzadmoghadam M , Mohammadi TM , Goudarzi R , Mohammadi M , Hasheminejad N . Is there a relationship between general and oral health‐related quality of life in partially edentulous patients before and after implant treatment? A quasi‐experimental study. Clin Oral Implants Res. 2020;31:557‐564. 32119138 10.1111/clr.13593

[41] Yeung CA . Effect of implant rehabilitation on oral health‐related quality of life with three different implant strategies. Evid Based Dent. 2020;21:92‐93. 32978537 10.1038/s41432-020-0112-8

[42] Fontijn‐Tekamp FA , Slagter AP , Van Der Bilt A , et al. Biting and chewing in overdentures, full dentures, and natural dentitions. J Dent Res. 2000;79:1519‐1524. 11005738 10.1177/00220345000790071501

[43] Fonteyne E , Matthys C , Bruneel L , Becue L , De Bruyn H , Van Lierde K . Articulation, oral function, and quality of life in patients treated with implant overdentures in the mandible: a prospective study. Clin Implant Dent Relat Res. 2021;23(3):388‐399. 33615684 10.1111/cid.12989

[44] Dellepiane E , Francesco P , Paola Z , Mugno MG , Pesce P , Menini M . Oral Health‐Related Quality of Life and full‐arch immediate loading rehabilitation: an evaluation of preoperative, intermediate, and posttreatment assessments of patients using a modification of the OHIP questionnaire. J Oral Implantol. 2020;46(6):540‐549. 33494102 10.1563/aaid-joi-D-19-00274

[45] Zhang Y , Chow L , Siu A , Fokas G , Chow TW , Mattheos N . Patient‐reported outcome measures (PROMs) and maintenance events in 2‐implant‐supported mandibular overdenture patients: A 5‐year prospective study. Clin Oral Implants Res. 2019;30:261‐276. 30714227 10.1111/clr.13412

[46] Doornewaard R , Gilbert M , Matthys C , Vervaeke S , Bronkhorst E , de Bruyn H . Improvement of quality of life with implant‐supported mandibular overdentures and the effect of implant type and surgical procedure on bone and soft tissue stability: a three‐year prospective split‐mouth trial. J Clin Med. 2019;8. 10.3390/jcm8060773 PMC6617188 31159202

[47] Yao CJ , Cao C , Bornstein MM , Mattheos N . Patient‐reported outcome measures of edentulous patients restored with implant‐supported removable and fixed prostheses: a systematic review. Clin Oral Implants Res. 2018;29(Suppl 16):241‐254. 30328202 10.1111/clr.13286

[48] Coutinho PC , Nogueira TE , Leles CR . Single‐implant mandibular overdentures: Clinical, radiographic, and patient‐reported outcomes after a 5‐year follow‐up. J Prosthet Dent. 2021. 10.1016/j.prosdent.2021.01.007 33640091

[49] Kutkut A , Bertoli E , Frazer R , Pinto‐Sinai G , Hidalgo RF , Studts J . A systematic review of studies comparing conventional complete denture and implant retained overdenture. J Prosthodont Res. 2018;62:1‐9. 28666845 10.1016/j.jpor.2017.06.004

[50] Sivaramakrishnan G , Sridharan K . Comparison of patient satisfaction with mini‐implant versus standard diameter implant overdentures: a systematic review and meta‐analysis of randomized controlled trials. Int J Implant Dent. 2017;3:29. 28669117 10.1186/s40729-017-0092-4 PMC5494032

[51] Sivaramakrishnan G , Sridharan K . Comparison of implant supported mandibular overdentures and conventional dentures on quality of life: a systematic review and meta‐analysis of randomized controlled studies. Aust Dent J. 2016;61:482‐488. 26836981 10.1111/adj.12416

[52] Allen PF , Thomason JM , Jepson NJA , Nohl F , Smith DG , Ellis J . A randomized controlled trial of implant‐retained mandibular overdentures. J Dent Res. 2006;85:547‐551. 16723653 10.1177/154405910608500613

[53] Allen PF , McMillan AS , Walshaw D . A patient‐based assessment of implant‐stabilized and conventional complete dentures. J Prosthet Dent. 2001;85:141‐147. 11208203 10.1067/mpr.2001.113214

[54] Heydecke G , Thomason JM , Lund JP , Feine JS . The impact of conventional and implant supported prostheses on social and sexual activities in edentulous adults Results from a randomized trial 2 months after treatment. J Dent. 2005;33:649‐657. 16139696 10.1016/j.jdent.2005.01.003

[55] Zembic A , Wismeijer D . Patient‐reported outcomes of maxillary implant‐supported overdentures compared with conventional dentures. Clin Oral Implants Res. 2014;25:441‐450. 23581398 10.1111/clr.12169

[56] Schuster AJ , da Rosa Possebon AP , Marcello‐Machado RM , Chagas‐Júnior OL , Faot F . Masticatory function and oral health‐related quality of life of patients with atrophic and non‐atrophic mandibles using implant‐retained mandibular overdentures: 3‐year results of a prospective clinical study. J Oral Rehabil. 2020;47:1278‐1286. 32772393 10.1111/joor.13072

[57] Fonteyne E , Burms E , Matthys C , Van Lierde K , De Bruyn H . Four‐implant‐supported overdenture treatment in the maxilla. Part II: Speech‐ and oral health‐related quality of life in patients with implant‐supported overdentures in the maxilla‐A prospective 3‐year follow‐up. Clin Implant Dent Relat Res. 2021;23(4):680‐691. 34378864 10.1111/cid.13034

[58] Garcia‐Minguillan G , Preciado A , Romeo M , Del Rio J , Lynch CD , Castillo‐Oyagüe R . Differences in self‐perceived OHRQoL between fully dentate subjects and edentulous patients depending on their prosthesis type, socio‐demographic profile, and clinical features. J Dent. 2021;29: 103756. 10.1016/j.jdent.2021.103756 34333055

[59] Kusumoto Y , Tanaka J , Miyoshi K , Higuchi D , Sato Y , Baba K . Impact of implant superstructure type on oral health‐related quality of life in edentulous patients. Clin Implant Dent Relat Res. 2020;22:319‐324. 32212313 10.1111/cid.12895

[60] Matthys C , Vervaeke S , Besseler J , Doornewaard R , Dierens M , De Bruyn H . Five years follow‐up of mandibular 2‐implant overdentures on locator or ball abutments: implant results, patient‐related outcome, and prosthetic aftercare. Clin Implant Dent Relat Res. 2019;21(5):835‐844. 31454159 10.1111/cid.12840

[61] Brandt S , Lauer HC , Fehrenz M , Güth JF , Romanos G , Winter A . Ball versus Locator® Attachments: a retrospective study on prosthetic maintenance and effect on oral‐health‐related quality of life. Materials. 2021;14(4):1051. 33672382 10.3390/ma14041051 PMC7926925

[62] Negoro M , Kanazawa M , Sato D , et al. Patient‐reported outcomes of implant‐assisted removable partial dentures with magnetic attachments using short implants: a prospective study. J Prosthodontic Res. 2021;64(4):554–558. 10.2186/jpr.JPR_D_20_00221 34193745

[63] Zhou H , Yang J , Ma CF , et al. Clinical outcomes of implant‐retained mandibular overdentures using the bar and magnetic attachment systems: an up to 5‐year retrospective study. Ann Transl Med. 2020;8(21):1360. 33313105 10.21037/atm-20-2531 PMC7723526

[64] Gündoğar H , Uzunkaya M , Öğüt S , Sari F . Effect of peri‐implant disease on oral health‐related quality of life in geriatric patients. Gerodontology. 2021. Online ahead of print. 10.1111/ger.12556 33977569

[65] Thomason JM , Heydecke G , Feine JS , Ellis JS . How do patients perceive the benefit of reconstructive dentistry with regard to oral health‐related quality of life and patient satisfaction? A systematic review. Clin Oral Implants Res. 2007;18(Suppl 3):168‐188. 17594380 10.1111/j.1600-0501.2007.01461.x

[66] Tsakos G , Steele JG , Marcenes W , Walls AW , Sheiham A . Clinical correlates of oral health‐related quality of life: evidence from a national sample of British older people. Eur J Oral Sci. 2006;114:391‐395. 17026504 10.1111/j.1600-0722.2006.00398.x

[67] Steele JG , Sanders AE , Slade GD , et al. How do age and tooth loss affect oral health impacts and quality of life? A study comparing two national samples. Community Dent Oral Epidemiol. 2004;32:107‐114. 15061859 10.1111/j.0301-5661.2004.00131.x

[68] Wong MC , McMillan AS . Tooth loss, denture wearing and oral health‐related quality of life in elderly Chinese people. Community Dent Health. 2005;3:156‐161. 16161879

[69] Kurosaki Y , Kimura‐Ono A , Mino T , et al. Six‐year follow‐up assessment of prosthesis survival and oral health‐related quality of life in individuals with partial edentulism treated with three types of prosthodontic rehabilitation. J Prosthodont Res. 2021;65(3):332‐339. 33281174 10.2186/jpr.JPR_D_20_00095

[70] Dong H , Zhou N , Liu H , et al. Satisfaction analysis of patients with single implant treatments based on a questionnaire survey. Patient Prefer Adherence. 2019;13:695‐704. 31190753 10.2147/PPA.S201088 PMC6519022

[71] AlZarea BK . Oral health related quality‐of‐life outcomes of partially edentulous patients treated with implant‐supported single crowns or fixed partial dentures. J Clin Exp Dent. 2017;9:e666‐e671. 28512544 10.4317/jced.53661 PMC5429479

[72] Gerritsen AE , Allen PF , Witter DJ , Bronkhorst EM , Creugers NH . Tooth loss and oral health‐related quality of life: a systematic review and meta‐analysis. Health Qual Life Outcomes. 2010;8:126. 21050499 10.1186/1477-7525-8-126 PMC2992503

[73] Alzarea BK . Assessment and Evaluation of Quality of Life (OHRQoL) of Patients with Dental Implants Using the Oral Health Impact Profile (OHIP‐14) ‐ A Clinical Study. J Clin Diagn Res. 2016;10(ZC57‐60):56. 10.7860/JCDR/2016/18575.7622 PMC4866251 27190953

[74] Sargozaie N , Moeintaghavi A , Shojaie H . Comparing the quality of life of patients requesting dental implants before and after implant. Open Dent J. 2017;11:485‐491. 29114333 10.2174/1874210601711010485 PMC5646019

[75] Reissmann DR , Dard M , Lamprecht R , Struppek J , Heydecke G . Oral health‐related quality of life in subjects with implant‐supported prostheses: a systematic review. J Dent. 2017;65:22‐40. 28789860 10.1016/j.jdent.2017.08.003

[76] Candel‐Marti E , Penarrocha‐Oltra D , Penarrocha‐Diago M , Penarrocha‐Diago M . Satisfaction and quality of life with palatal positioned implants in severely atrophic maxillae versus conventional implants supporting fixed full‐arch prostheses. Med Oral Patol Oral Cir Bucal. 2015;20:e751‐756. 26116847 10.4317/medoral.20706 PMC4670257

[77] Torres BL , Costa FO , Modena CM , Cota LOM , Côrtes MIS , Seraidarian PI . Association between personality traits and quality of life in patients treated with conventional mandibular dentures or implant‐supported overdentures. J Oral Rehabil. 2011;38:454‐461. 21039749 10.1111/j.1365-2842.2010.02165.x

[78] Yu S‐J , Chen P , Zhu G‐X . Relationship between implantation of missing anterior teeth and oral health‐related quality of life. Qual Life Res. 2013;22:1613‐1620. 23161328 10.1007/s11136-012-0314-4

[79] Wang Y , Bäumer D , Ozga A‐K , Körner G , Bäumer A . Patient satisfaction and oral health‐related quality of life 10 years after implant placement. BMC Oral Health. 2021;21:30. 33446161 10.1186/s12903-020-01381-3 PMC7807859

[80] Furhauser R , Florescu D , Benesch T , Haas R , Mailath G , Watzek G . Evaluation of soft tissue around single‐tooth implant crowns: the pink esthetic score. Clin Oral Implants Res. 2005;16:639‐644. 16307569 10.1111/j.1600-0501.2005.01193.x

[81] Meijer HJ , Stellingsma K , Meijndert L , Raghoebar GM . A new index for rating aesthetics of implant‐supported single crowns and adjacent soft tissues–the Implant Crown Aesthetic Index. Clin Oral Implants Res. 2005;16:645‐649. 16307570 10.1111/j.1600-0501.2005.01128.x

[82] Vaidya S , Ho YL , Hao J , Lang NP , Mattheos N . Evaluation of the influence exerted by different dental specialty backgrounds and measuring instrument reproducibility on esthetic aspects of maxillary implant‐supported single crown. Clin Oral Implants Res. 2015;26:250‐256. 25496129 10.1111/clr.12532

[83] Chang M , Odman PA , Wennstrom JL , Andersson B . Esthetic outcome of implant‐supported single‐tooth replacements assessed by the patient and by prosthodontists. Int J Prosthodont. 1999;12:335‐341. 10635203

[84] Esposito M , Grusovin MG , Worthington HV . Agreement of quantitative subjective evaluation of esthetic changes in implant dentistry by patients and practitioners. Int J Oral Maxillofac Implants. 2009;24:309‐315. 19492647

[85] Wittneben JG , Wismeijer D , Brägger U , Joda T , Abou‐Ayash S . Patient‐reported outcome measures focusing on aesthetics of implant‐ and tooth‐supported fixed dental prostheses: a systematic review and meta‐analysis. Clin Oral Implants Res. 2018;29(Suppl):16. 10.1111/clr.13295 30328183

[86] Hosseini M , Gotfredsen K . A feasible, aesthetic quality evaluation of implant‐supported single crowns: an analysis of validity and reliability. Clin Oral Implants Res. 2012;23:453‐458. 21443589 10.1111/j.1600-0501.2011.02162.x

[87] Hosseini M , Worsaae N , Schiødt M , Gotfredsen K . 3‐year prospective study of implant‐ supported, single‐tooth restorations of all‐ceramic and metal‐ceramic materials in patients with tooth agenesis. Clin Oral Implants Res. 2013;24:1078‐1087. 22708959 10.1111/j.1600-0501.2012.02514.x

[88] Jemt T . Regeneration of gingival papillae after single‐implant treatment. Int J Periodontics Restorative Dent. 1997;17:326‐333. 9497723

[89] Schropp L , Isidor F , Kostopoulos L , Wenzel A . Interproximal papilla levels following early versus delayed placement of single‐tooth implants: a controlled clinical trial. Int J Oral Maxillofac Implants. 2005;20:753‐761. 16274150

[90] Thoma DS , Ioannidis A , Cathomen E , Hämmerle CHF , Hüsler J , Jung RE . Discoloration of the peri‐implant mucosa caused by zirconia and titanium implants. Int J Periodontics Restorative Dent. 2016;36:39‐45. 10.11607/prd.2663 26697552

[91] Sailer I , Fehmer V , Ioannidis A , Hammerle CH , Thoma DS . Threshold value for the perception of color changes of human gingiva. Int J Periodontics Restorative Dent. 2014;34:757‐762. 25411730 10.11607/prd.2174

[92] Belser UC , Grütter L , Vailati F , Bornstein MM , Weber H‐P , Buser D . Outcome evaluation of early placed maxillary anterior single‐tooth implants using objective esthetic criteria: a cross‐sectional, retrospective study in 45 patients with a 2‐ to 4‐year follow‐up using pink and white esthetic scores. J Periodontol. 2009;80:140‐151. 19228100 10.1902/jop.2009.080435

[93] MacEntee MI , Walton JN . The economics of complete dentures and implant‐related services: a framework for analysis and preliminary outcomes. J Prosthet Dent. 1998;79:24‐30. 9474537 10.1016/s0022-3913(98)70189-1

[94] Brägger U , Krenander P , Lang NP . Economic aspects of single‐tooth replacement. Clin Oral Implants Res. 2005;16:335‐341. 15877754 10.1111/j.1600-0501.2005.01112.x

[95] Drummond MF , Sculpher MJ , Torrance GW , O’Brien BJ , Stoddart GL . Methods for the Economic Evaluation of Health Care Programmes, 3rd ed. Oxford University Press; 2005.

[96] Hettiarachchi RM , Kularatna S , Downes M , et al. The cost‐effectiveness of oral health interventions: a systematic review of cost‐utility analyses. Community Dent Oral Epidemiol. 2018;46. 10.1111/cdoe.12336 28925508

[97] Jensen C , Ross J , Feenstra T , et al. Cost‐effectiveness of implant‐supported mandibular removable partial dentures. Clin Oral Implants Res. 2017;28:594‐601. 27080041 10.1111/clr.12840

[98] Palmqvist S , Owall B , Schou S . A prospective randomized clinical study comparing implant‐supported fixed prostheses and overdentures in the edentulous mandible: prosthodontic production time and costs. Int J Prosthodont. 2004;17:231‐235. 15119877

[99] Ravidà A , Barootchi S , Tattan M , Saleh MHA , Gargallo‐Albiol J , Wang H‐L . Clinical outcomes and cost effectiveness of computer‐guided versus conventional implant‐retained hybrid prostheses: A long‐term retrospective analysis of treatment protocols. J Periodontol. 2018;89:1015‐1024. 29761505 10.1002/JPER.18-0015

[100] Ravidà A , Tattan M , Askar H , Barootchi S , Tavelli L , Wang H‐L . Comparison of three different types of implant‐supported fixed dental prostheses: a long‐term retrospective study of clinical outcomes and cost‐effectiveness. Clin Oral Implants Res. 2019;30:295‐305. 30758878 10.1111/clr.13415

[101] Sanz M , Herrera D , Kebschull M , et al. Treatment of stage I‐III periodontitis‐The EFP S3 level clinical practice guideline. EFP Workshop Participants and Methodological Consultants. J Clin Periodontol. 2020;Suppl 22:4‐60. 10.1111/jcpe.13290 PMC7891343 32383274

